# Intracellular trafficking of begomoviruses in the midgut cells of their insect vector

**DOI:** 10.1371/journal.ppat.1006866

**Published:** 2018-01-25

**Authors:** Wen-Qiang Xia, Yan Liang, Yao Chi, Li-Long Pan, Jing Zhao, Shu-Sheng Liu, Xiao-Wei Wang

**Affiliations:** Ministry of Agriculture Key Laboratory of Molecular Biology of Crop Pathogens and Insects, Institute of Insect Sciences, Zhejiang University, Hangzhou, China; Agriculture and Agri-Food Canada, CANADA

## Abstract

Begomoviruses are exclusively transmitted by whiteflies in a persistent circulative manner and cause considerable economic losses to crop production worldwide. Previous studies have shown that begomoviruses accumulate in vesicle-like structures in whitefly midgut cells and that clathrin-mediated endocytosis is responsible for their internalization. However, the process by which begomoviruses are trafficked within whitefly midgut cells remains largely unknown. In this study, we investigated the roles of vesicle trafficking in the transport of *Tomato yellow leaf curl virus* (TYLCV), a begomovirus that has spread to over 50 countries and caused extensive damage to a range of important crops, within midgut cells of whitefly (*Bemisia tabaci*). By disrupting vesicle trafficking using RNA silencing and inhibitors, we demonstrated that the early steps of endosomal trafficking are important for the intracellular transport of TYLCV in the whitefly midgut. In addition, our data show that, unlike many animal viruses, TYCLV is trafficked within cells in a manner independent of recycling endosomes, late endosomes, lysosomes, the Golgi apparatus and the endoplasmic reticulum. Instead, our results suggest that TYLCV might be transported directly from early endosomes to the basal plasma membrane and released into the hemolymph. Silencing of the sorting nexin Snx12, which may be involved in membrane tubulation, resulted in fewer viral particles in hemolymph; this suggests that the tubular endosomal network may be involved in the transport of TYLCV. Our results also support a role for the endo-lysosomal system in viral degradation. We further showed that the functions of vector early endosomes and sorting nexin Snx12 are conserved in the transmission of several other begomoviruses. Overall, our data indicate the importance of early endosomes and the tubular endosomal network in begomovirus transmission.

## Author summary

Many plant viruses are vectored by insects in a persistent circulative manner. In this process, the transport of virus from the gut lumen into the hemolymph of the vector is an important step. Identification of vector components involved in this transport process could lead to new strategies to combat virus spread. Here, we demonstrate that the early steps of endosomal trafficking are important for the transport of begomoviruses across epithelial cells in the whitefly midgut. After internalization by midgut epithelial cells, begomoviruses are transported directly from early endosomes to the basal plasma membrane in a fast recycling route. We also identified several host processes that are involved in begomovirus transmission, including vesicle transport and membrane fusion events that promote virus trafficking. To our knowledge, this is the first report on mechanisms governing the intracellular transport of begomoviruses in their insect vector. Our study provides new insights into the transmission mechanisms of circulative plant viruses and gives direction to future research into begomovirus transmission blocking strategies.

## Introduction

Insects transmit the majority of plant viruses [1–3]. The process of virus transmission by an insect vector varies based on how the virus is acquired, retained, and inoculated into plants. Some plant viruses, such as members of the genera *Caulimovirus*, *Cucumovirus* and *Potyvirus*, are transmitted in a non-circulative manner, in which the viruses are acquired by feeding on infected plants, retained on the surface of the stylet, food canal, or foregut, and then inoculated into new host plants [4–6]. The coat proteins or non-structural proteins of some of these viruses aid their retention on the inner cuticular lining of the vector feeding apparatus via interactions with specific receptors [7–9]. By contrast, persistent circulative viruses have developed much more complex interactions with their vectors. These viruses need to travel from the gut lumen into the hemolymph, move to the salivary gland, and finally be secreted from the salivary gland into new host plants [10, 11]. Barriers to this transmission exist in the midgut and salivary gland, in which septate junctions are formed among epithelial cells to control the exchange of substances between hemolymph and midgut or salivary gland [12]. In many cases, circulative viruses can be transmitted at higher rates after injection into the vector hemocoel than after oral acquisition, suggesting that delivery across the insect gut is a considerable barrier to virus transmission [13, 14].

Understanding the cellular processes that viruses exploit to facilitate their transport within vectors remains challenging. Animal viruses enter cells either via direct penetration through the plasma membrane or via endocytosis. The majority of animal viruses enter cells by hijacking the host cell’s clathrin-mediated endocytosis pathway [15]. Viral particles are then transported to early endosomes and sorted to various intracellular destinations [15, 16]. After arrival in the lumen of specific vesicles, environmental cues such as acidified pH, changes in the redox environment, and proteolytic cleavage induce conformational changes in viral proteins and activate viral penetration into cytoplasm [17–19]. For instance, a significant proportion of Adeno-associated virus (AAV) particles are transported to the trans-Golgi network (TGN), whereas Ebola virus particles need to be transported from endosomes to lysosomes to complete their infection cycle [20, 21]. However, the intracellular routes plant viruses take within their vectors may differ significantly from those animal viruses take. How insect-vectored plant viruses exploit vector cells for delivery across the midgut is poorly understood.

In the past three decades, begomoviruses (genus *Begomovirus*, family Geminiviridae), one of the most important groups of plant viruses in tropical and subtropical regions, have caused considerable economic losses to a variety of crops [3, 22–24]. Begomoviruses are transmitted exclusively by the whitefly *Bemisia tabaci* in a persistent circulative manner [3], and the emergence of begomoviruses as important pathogens is closely related to the increasing prevalence of whiteflies worldwide [25, 26]. A previous immuno-electron microscopy study using gold-labeled secondary antibodies showed that the begomovirus *Tomato yellow leaf curl virus* (TYLCV) can be detected in vesicle-like structures in the midgut cells of whitefly [27]. Pan *et al*. [28] further showed that TYLCV can be internalized into midgut cells through the clathrin-dependent pathway and that the endosomal system may play an important role in virus transport across the whitefly midgut. These results suggest that membranous vesicles are recruited by begomoviruses for their entry into vector midgut cells.

However, the process by which these viruses are trafficked in epithelial cells remains largely unknown. Many questions, including how begomoviruses are transported to the basal membrane of the epithelial cell and secreted into hemolymph, have not been answered. In this study, we explored begomovirus trafficking in whitefly midgut cells using immunofluorescence, inhibitors, and double strand RNA (dsRNA)-mediated RNA silencing. We showed that only early steps of endosomal trafficking are involved in the intracellular transport of begomoviruses, suggesting that viral particles might be transported directly from early endosomes to basal membranes.

## Results

### Importance of vesicle trafficking in TYLCV transport

**Fig 1:**
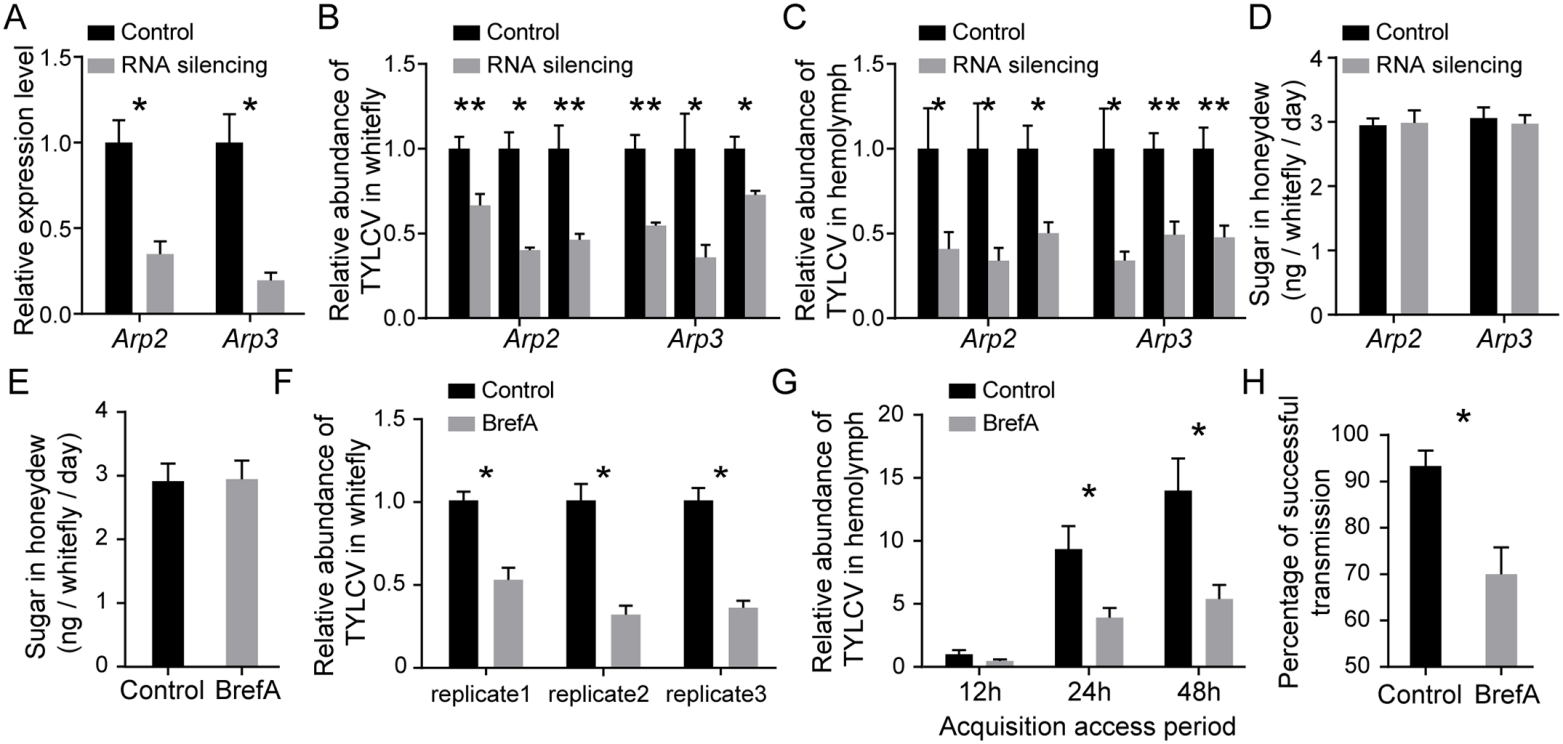
Inhibiting TYLCV acquisition by disrupting vesicle traffic. (A-C) Silencing of *Arp 2* and *Arp3*. Genes were silenced by injecting dsRNA into hemolymph of whitefly. Whiteflies injected with dsRNA of GFP were included as a control. The insects were then kept on cotton plants. (A) Seventy-two hours after dsRNA injection, gene expression levels were measured by quantitative RT-PCR (n = 4–5, n represents the number of samples). (B-C) The effect of the Arp2/3 complex on virus acquisition. Seventy-two hours after dsRNA injection, the treatment group and its control group were caged on two symmetrical leaves of the same TYLCV-infected plants. The abundance of virus in the whitefly whole body (B) and hemolymph (C) was measured after a 48 h acquisition access period (AAP) by quantitative PCR and compared with that in controls injected with dsRNA of GFP (for the whole body of whitefly, n = 4–8; for hemolymph, n = 8–24). (D-E) The quantity of honeydew excreted by whitefly. After RNA silencing or inhibitor feeding and 48 h AAP on tomato plant, the honeydew of whiteflies was collected from the surface of leaves and micro-cages and the content of sugar per whitefly per day was quantified (n = 5). (F) Effect of BrefA on virus acquisition. Whiteflies were fed with BrefA for 24 h and kept on TYLCV-infected plants for 48 h. Then, the abundance of virus in the whitefly whole body was measured by quantitative PCR and compared with that in ethanol-fed controls (n = 4). (G) Whiteflies were allowed to feed on TYLCV-infected plants for various AAP after feeding with BrefA or solvent ethanol. The abundance of virus in hemolymph was measured by quantitative PCR (n = 8–11). (H) Effect of BrefA on virus transmission. Whiteflies were first fed with BrefA and then transferred to TYLCV-infected plants for 12 h AAP. Next, the whiteflies were allowed to feed on tomato seedlings for 48 h and then removed. Percentages of tomato plants infected by the virus were calculated (n = 30). Data shown are mean ± SE. Statistically significant differences are indicated as *, P<0.05; **, P<0.01.

Animal viruses make extensive use of vesicle trafficking following endocytosis in their infection processes [15, 17]. The Arp2/3 complex can facilitate endocytosis and vesicle trafficking through its functions in organizing actin filaments and regulating polymerization [29–31]. To confirm their roles in the transmission of TYLCV by whitefly, *Arp2* and *Arp3* were subjected to dsRNA-mediated gene silencing analysis. The efficiency of dsRNA-mediated gene silencing was confirmed at the mRNA level by quantitative RT-PCR (Fig 1A). Silencing of *Arp2* or *Arp3* resulted in significant decreases in the quantity of TYLCV acquired by whiteflies (Fig 1B). Next, whether Arp2 and Arp3 influenced the transport of viral particles across the midgut barrier was examined by quantifying virus abundance in the hemolymph. Virus abundance in hemolymph was significantly lower in the treatment groups than in the control groups (Fig 1C). To rule out the possibility that silencing *Arp2* and *Arp3* influences the ingestion of virus-containing phloem sap by whiteflies, the honeydew of whiteflies was collected and its total sugar content was quantified by the anthrone reaction. No significant difference was observed between the control and treatment groups (Fig 1D), suggesting that silencing *Arp2* or *Arp3* did not affect virus ingestion.

To further investigate the role of vesicle trafficking in TYLCV transmission, we fed whiteflies with Brefeldin A (BrefA), which can disrupt intracellular vesicle transport by a pleiotropic effect on the entire endosome system and the Golgi apparatus [32]. BrefA treatment induced the mislocalization of the trans-Golgi network (S1 Fig), confirming its effects in the whiteflies, and BrefA treatment did not affect whitefly feeding (Fig 1E). The acquisition of TYLCV by whitefly was significantly inhibited after BrefA treatment (Fig 1F). Feeding of BrefA inhibited the transport of virus across the midgut and led to a significant reduction in hemolymph virus abundance after 24 and 48 hours acquisition access period (AAP) on an infected plant (Fig 1G). Moreover, when whiteflies were used to transmit TYLCV from infected to uninfected host plants, BrefA treatment significantly decreased their transmission efficiency (Fig 1H). Overall, our data suggest that vesicle trafficking is critical for the transport of TYLCV into vector hemolymph.

### Localization of TYLCV in midgut cells

**Fig 2:**
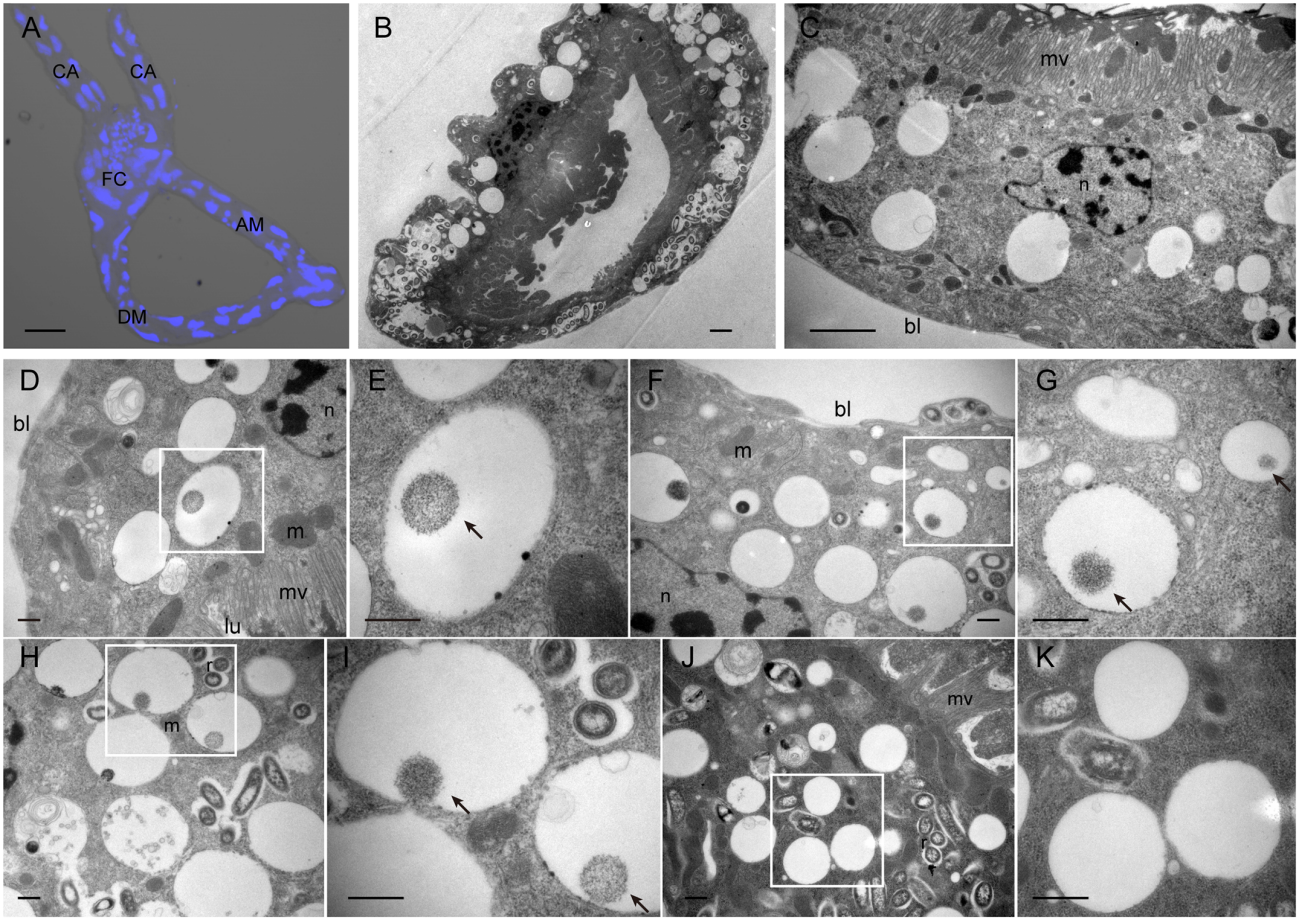
Overview and electron microscopy observations of whitefly midgut. (A) Dissected alimentary canal of whitefly *B*. *tabaci*, showing the filter chamber (FC), ascending midgut (AM), descending midgut (DM) and gastric caecum (CA). Blue signal indicates nuclei of cells. Scale bar 50 μm. (B) Electron micrograph of midgut cross section. Scale bar 2 μm. (C) Image of midgut exhibiting epithelial cells and microvilli (*mv*); *bl* basal lamina. Scale bar 2 μm. (D-K) Midguts of whiteflies exposed to TYLCV-infected tomato plants for a 7 d AAP (D-I) or of control whiteflies that fed on uninfected plants (J-K) were osmium-fixed and prepared for electron microscopy. Large paracrystalline accumulations of TYLCV-like particles can be observed in the apical region close to microvilli (*mv*) (D), close to the basal lamina (*bl*) (H), or between these locations (F). Arrows indicate paracrystalline accumulations of TYLCV-like particles which were always found enclosed by membrane. The membranous vesicles in control whiteflies are devoid of TYLCV-like particles (J). Panels E, G, I, and K show the enlarged images. *bl* basal lamina, *m* mitochondrion, *n* nucleus, *lu* midgut lumen, *r* Rickettsia. Scale bar 500 nm.

The structure of the whitefly alimentary canal and filter chamber are shown in Fig 2A, and the two arms that join the filter chamber are gastric caeca [33]. Fig 2B and 2C show cross sections of the midgut. The whitefly midgut has only one layer of epithelial cells and is rich in microvilli. In thin sections of whiteflies exposed to the virus for a 7d AAP, aggregates of TYLCV-like particles, ca. 19 nm in diameter, were consistently found in epithelial cells and were bound by a single membrane (Fig 2D–2I). These accumulations were mostly found in paracrystalline arrays, which is similar to the form Maize streak virus (MSV) takes in its leafhopper vector [34]. The vesicles containing these aggregates were not limited to any particular region of epithelial cells, but could be found in the apical region close to microvilli (Fig 2D and 2E), close to the basal plasma membrane (Fig 2F and 2G), or between these locations (Fig 2H and 2I). In control whiteflies, TYLCV-like particle aggregates were never found in midgut cells (Fig 2J and 2K). However, whether these aggregates consisted of TYLCV particles remains uncertain. Subsequently, we used immunofluorescence to localize the virus in the midgut, in relation to lectins used as organelle markers.

Lectins are oligomeric proteins with saccharide-binding sites that can recognize and bind particular sugar molecules. Usually, specific oligosaccharides are associated with a certain organelle, and lectins can thus serve to identify cellular components. Lectin WGA binds to *N*-acetylglucosaminyl residues and is used for staining the nuclear core, plasma membrane and sarcolemma [35–37]. Lectin GS-II is used for staining intermediate-to-trans Golgi because of its affinity for α- and β-*N*-acetyl-D-glucosaminyl residues [38]. Lectin HPA selectively binds to type A erythrocytes and to α-*N*-acetylgalactosaminyl residues found in the cis-Golgi [39].

**Fig 3:**
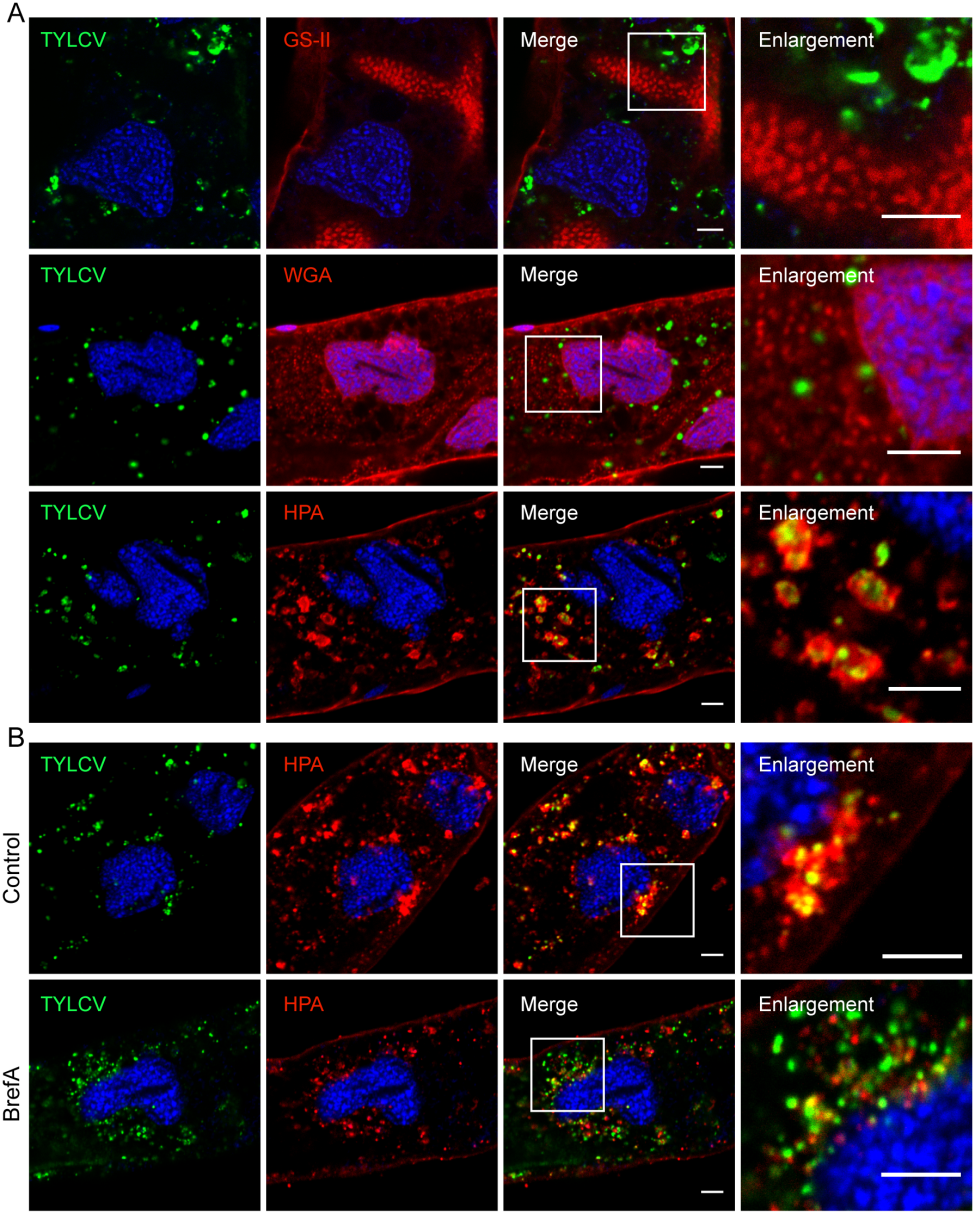
Subcellular localization of TYLCV in the epithelial cells of whitefly midgut. (A) Confocal microscopy images showing the colocalization of TYLCV with HPA-labeled structures. Midguts of whiteflies exposed to TYLCV-infected tomato plants for a 3 d AAP were dissected and prepared for immunofluorescence. TYLCV was identified using a specific antibody against its coat protein, the only known structural protein of TYLCV. Lectins GS-II, HPA, and WGA were used to label different kinds of glycoproteins. Colocalization between TYLCV and GS-II, WGA, and HPA-labeled structures were quantified as 0.04 ± 0.02, 0.08 ± 0.01, and 0.43 ± 0.02, respectively (Pearson’s coefficient, mean ± SE, n = 14–20). Scale bar, 5 μm. (B) Whiteflies that had been fed with BrefA and control whiteflies that had been fed with solvent ethanol were transferred to TYLCV-infected plants for 3 d AAP. BrefA disrupted the colocalization between TYLCV and HPA. Colocalization between TYLCV and HPA in whiteflies fed with solvent or BrefA were quantified as 0.48 ± 0.02 and 0.20 ± 0.02, respectively (Pearson’s coefficient, mean ± SE, n = 10). Scale bar, 5 μm. Representative images that were used to generate the data are provided in S6 Fig.

The midgut of whiteflies that had a 72 h AAP on TYLCV-infected plants were dissected and prepared for immunofluorescence. Confocal microscopy showed no appreciable colocalization between TYLCV and WGA or GS-II (Fig 3A). However, HPA lectin specifically labelled spherical structures within midgut cells, and almost all TYLCV colocalized with the HPA-labeled structures. In the enlarged image, HPA lectin labels a vesicle-like structure surrounding TYLCV (Fig 3A).

Previous studies showed that BrefA can impair the functions of various vesicles [20, 32]. We next investigated the effect of BrefA on the HPA-labeled structures and TYLCV. Whiteflies that had been fed with BrefA were transferred to TYLCV-infected plants for 72 h and prepared for immunofluorescence. BrefA had no visible effect on the formation of HPA-labeled structures; however, it disrupted the colocalization between HPA-labeled structures and TYLCV (Fig 3B). This indicates that BrefA treatment can lead to disrupted localization of TYLCV.

**Fig 4:**
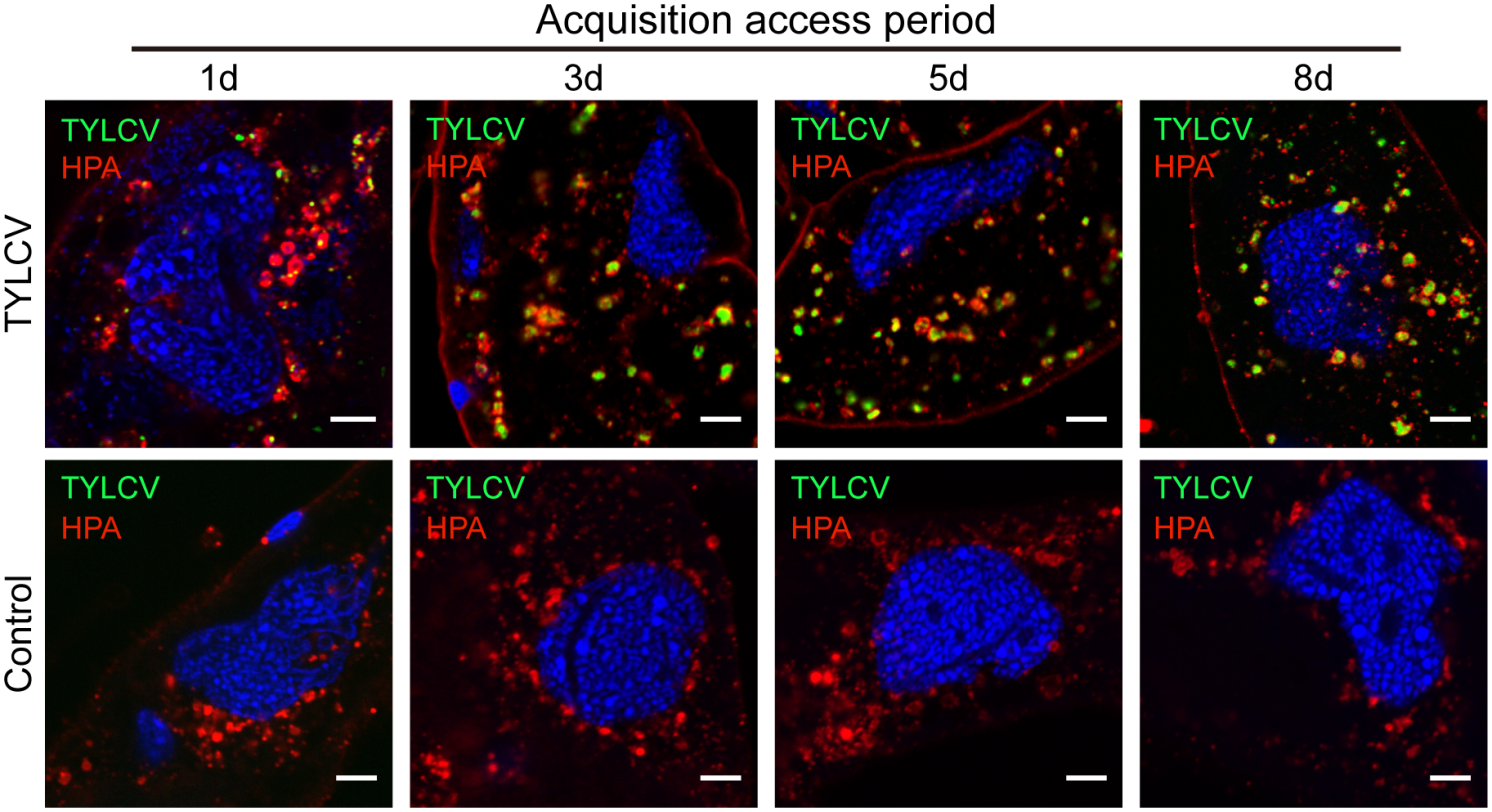
Time scale localization of TYLCV in the epithelial cells of whitefly midgut. Whiteflies were allowed to feed on TYLCV-infected plants for different AAP and prepared for immunofluorescence. Whiteflies that had fed on uninfected tomato plants were included as a negative control. At the four time points, colocalization between TYLCV and HPA was quantified as 0.24 ± 0.01, 0.44 ± 0.02, 0.44 ± 0.02 and 0.50 ± 0.05, respectively (Pearson’s coefficient, mean ± SE, n = 13–20). Scale bar, 5 μm. Representative images that were used to generate the data are provided in S7 Fig.

Next, whiteflies were prepared for immunofluorescence after different AAP on TYLCV-infected plants. The accumulation of virus in HPA-labeled structures was observed as early as 1 day after transfer to infected plants. The quantity of virus in midgut cells gradually increased over time, and most viruses were localized within HPA-labeled vesicles from day 1 to day 8 (Fig 4). The strict colocalization between viral particles and HPA-labeled vesicles indicates that the majority of TYLCV accumulated in a single kind of vesicle and few (if any) particles were released into the cytoplasm. Since it has been reported that the terminal α-*N*-acetylgalactosaminyl residues that lectin HPA selectively binds to are added in the cis-Golgi and then substituted in the trans-Golgi [39], our results suggest that, after internalization, TYLCV might localize in the Golgi apparatus.

### Intracellular transport of TYLCV does not depend on the Golgi apparatus

**Fig 5:**
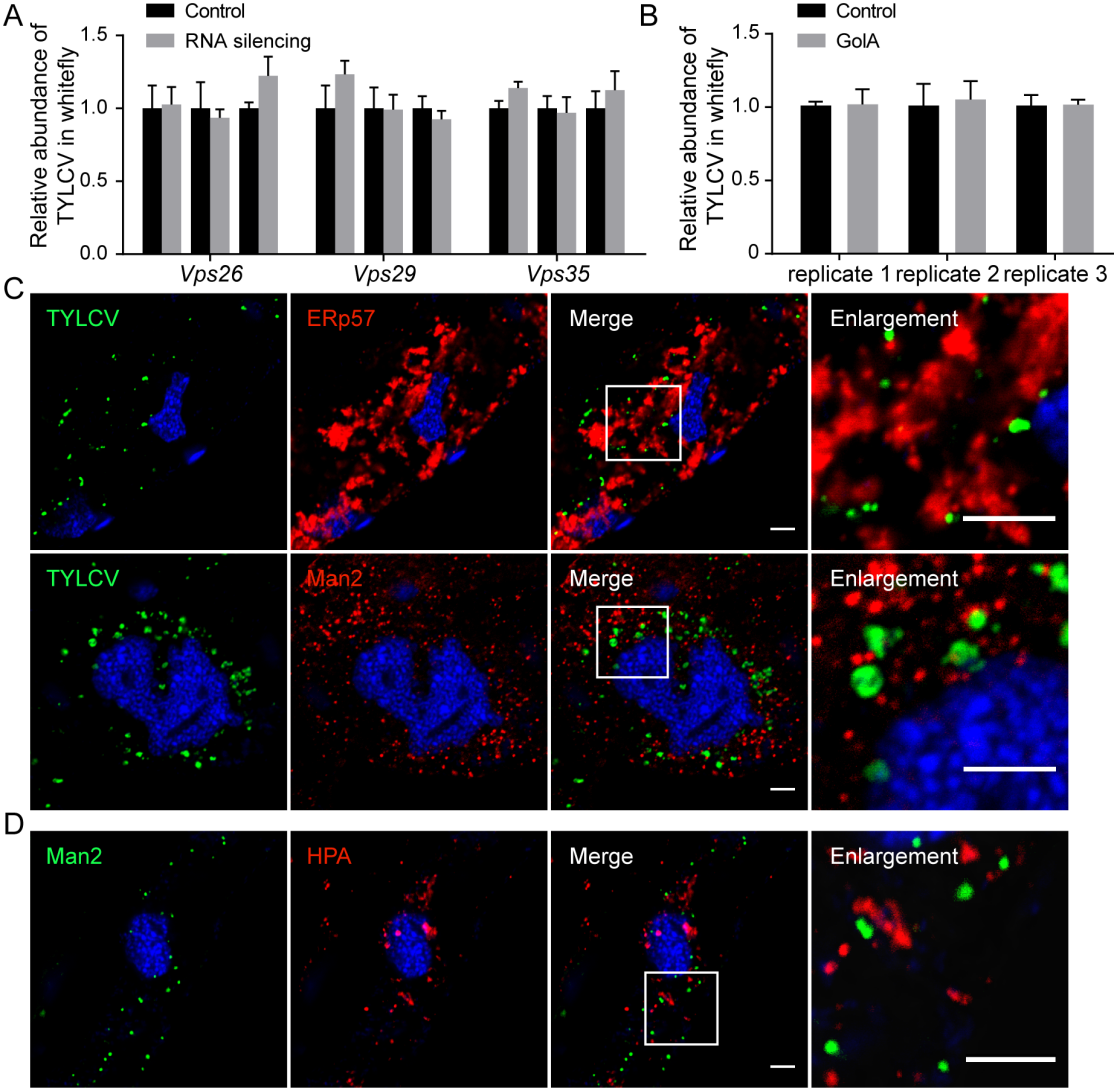
Lack of evidence for the involvement of Golgi apparatus in TYLCV trafficking. (A) TYLCV trafficking was not influenced by silencing Golgi apparatus-related genes. After gene silencing, whiteflies were transferred to TYLCV-infected plants for virus acquisition. The abundance of virus in the whiteflies was quantified 48 h later and compared with that in controls injected with dsRNA of GFP (n = 4–7). (B) Effect of GolA on virus acquisition. Whiteflies were fed with GolA for 24 h to disrupt the function of the Golgi apparatus and transferred to TYLCV-infected plants. The abundance of virus in the whiteflies was measured after 48 h by quantitative PCR and compared with that in solvent-fed controls (n = 4). (C) Midguts of whiteflies exposed to TYLCV-infected tomato plants for a 3 d AAP were dissected and prepared for immunofluorescence. The ER and Golgi apparatus were identified using antibodies against ERp57 and alpha-mannosidase 2 (Man2), respectively. Colocalization between TYLCV and ERp57 or Man2 was quantified as 0.05 ± 0.01, 0.03 ± 0.01, respectively (Pearson’s coefficient, mean ± SE, n = 17–33). Scale bar, 5 μm. (D) The midguts of whiteflies were dissected and labeled by HPA lectin and antibody against Man2. Their colocalization was quantified as 0.10 ± 0.01 (Pearson’s coefficient, mean ± SE, n = 10). Scale bar, 5 μm. Representative images that were used to generate the data are provided in S8 Fig. Data shown are mean ± SE.

Retromer complex plays important roles in retrograde transport of cargoes to the Golgi apparatus, and its subunits Vps26, Vps29 and Vps35 are responsible for cargo selection [40]. To verify the role of the Golgi apparatus in TYLCV transport, these genes were selected as targets for RNA interference, and we also used Golgicide A (GolA), a highly specific inhibitor of the Golgi apparatus. Quantitative RT-PCR showed that *Vps26*, *Vps29* and *Vps35* were successfully silenced (S2 Fig). The effect of GolA on the Golgi apparatus was confirmed by the dispersal of the trans-Golgi network (S2 Fig). Measurement of sugar content in honeydew indicated that silencing of these genes or GolA treatment did not influence whitefly feeding (S2 Fig). However, to our surprise, neither GolA treatment nor silencing of retromer complex genes inhibited virus acquisition by whitefly (Fig 5A and 5B), suggesting that the Golgi apparatus may not be involved in the transport of virus.

This result is surprising because retromer complex is essential for retrograde transport to the Golgi, and TYLCV was found to be colocalized strictly with an HPA-labeled structure, which is predicted to be the cis-Golgi apparatus [40, 41]. To address this apparent discrepancy, midguts of virus-carrying whiteflies were further labeled using antibodies against marker proteins for the Golgi apparatus and endoplasmic reticulum (ER) [42]. Surprisingly, no appreciable colocalization was detected between virus and the Golgi apparatus or ER (Fig 5C). We further investigated the spatial relationship between the Golgi apparatus marker protein and HPA-labeled structures and found no appreciable colocalization (Fig 5D). Taken together, our results suggest that TYLCV virus particles accumulated in some kind of vesicle labelled by HPA, but not in the Golgi apparatus or ER.

### Accumulation of viral particles in endosomes or lysosomes

To investigate what kind of vesicle this is, we conducted lectin affinity capture followed by mass spectrometry to identify proteins on virus-containing vesicles labeled by lectin HPA. Five proteins were captured by lectin HPA: glycosylphosphatidylinositol (GPI)-anchored glycoprotein, myosin heavy chain, beta-actin, lysosomal associated membrane protein-1 (Lamp1) and sarcoplasmic/endoplasmic reticulum calcium-transporting ATPase (SERCA). GPI-anchored glycoprotein is localized mainly on the plasma membrane but can be endocytosed via caveolae or other plasma membrane components and then transported to endosomes [43]. The cytoskeleton and motor proteins are critical to vesicular movement, localization, and membrane fission. We speculated that myosin heavy chain and beta-actin were detected due to their interactions with proteins on vesicles, not their direct glycosylation [44, 45]. Lamp1 is a glycoprotein that is primarily associated with the endosome and the lysosome, and SERCA may be responsible for calcium entry into lysosomes [46, 47]. This suggests that viral particles may accumulate in endosomes or lysosomes following their internalization.

### Early endosomes are essential for TYLCV transport

**Fig 6:**
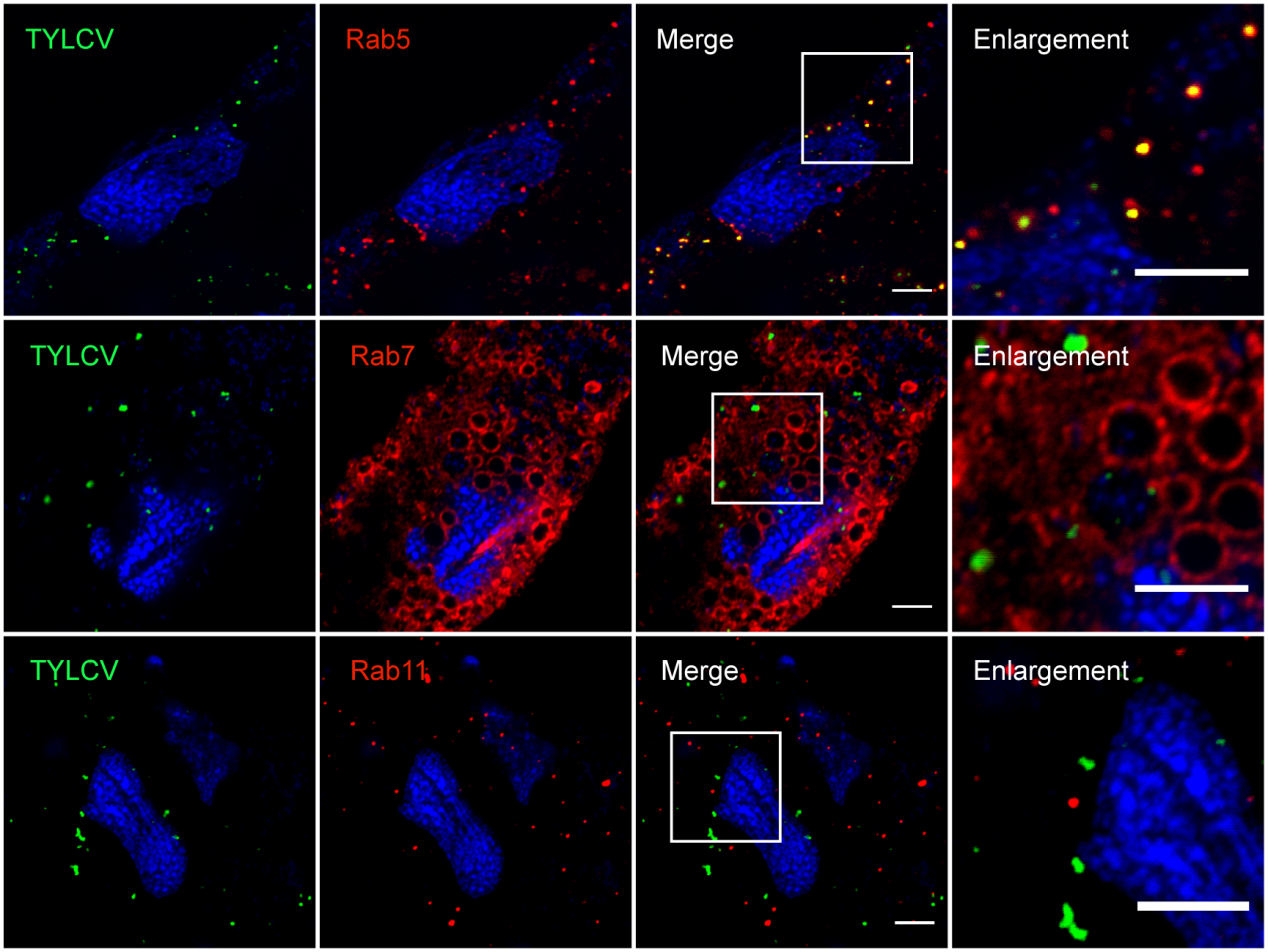
Colocalization of TYLCV and endosomes. Midguts of whiteflies exposed to TYLCV-infected tomato plants for a 3 d AAP were dissected and prepared for immunofluorescence. Antibodies against Rab5, Rab7 and Rab11 were used to label early endosomes, late endosomes and recycling endosomes, respectively. Colocalization between TYLCV and these structures were quantified as 0.36 ± 0.05, 0.05 ± 0.01, 0.04 ± 0.01, respectively (Pearson’s coefficient, mean ± SE, n = 11–15). Scale bar, 5 μm. Representative images that were used to generate the data are provided in S9 Fig.

To determine the roles of endosomes in virus trafficking, we first examined whether TYLCV localizes within endosomes using specific antibodies against marker proteins Rab5 (early endosome), Rab7 (late endosome) and Rab11 (recycling endosome). We found that some TYLCV particles were colocalized with Rab5-labeled early endosomes (Fig 6A). However, we found no appreciable colocalization between TYLCV and Rab7 or Rab11 (Fig 6B and 6C).

**Fig 7:**
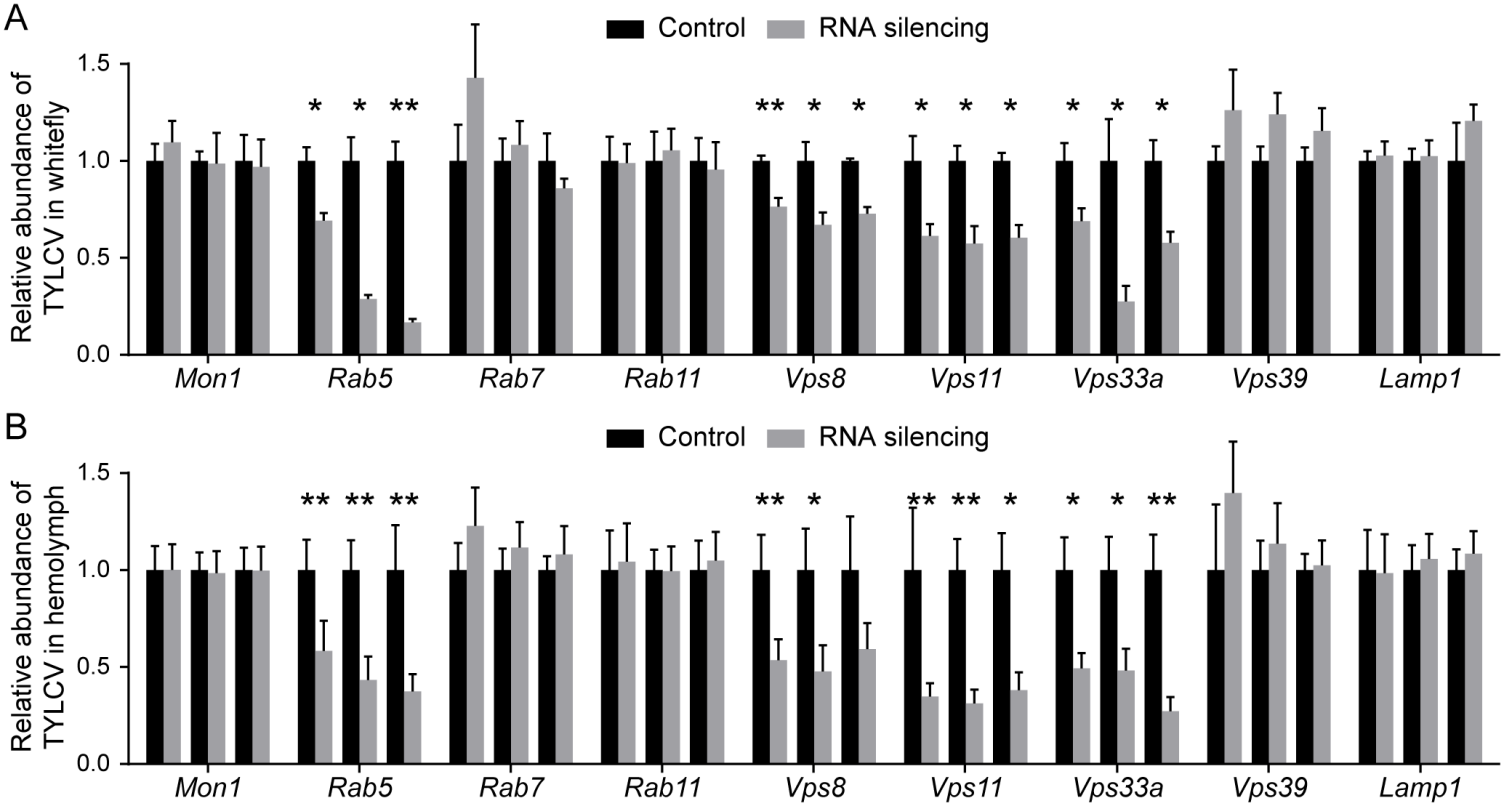
Involvement of endosomes in TYLCV trafficking. (A-B) TYLCV trafficking was influenced by knockdown of endosomal genes. Genes related to endosomal trafficking were silenced by dsRNA injection. The abundance of virus in the whitefly whole body (A) and hemolymph (B) were quantified by qRT-PCR after 48 h feeding on TYLCV-infected plants and compared with that in controls injected with dsRNA of GFP (for the whitefly whole body, n = 4–8; for hemolymph, n = 7–24). Data shown are mean ± SE. Statistically significant differences are indicated as: *, P<0.05; **, P<0.01.

Knock down of *Rab5*, *Vps8* (a specific subunit of the endosomal CORVET complex), *Vps11* and *Vps33a* (two subunits shared by CORVET and the HOPS complex) suppressed the acquisition of virus and inhibited the delivery of TYLCV across the midgut (Fig 7). Whereas silencing *Mon1*, *Rab7*, *Rab11*, *Lamp1*, *and Vps39* (a specific subunit of the HOPS complex) had no influence on the virus titer in whitefly (Fig 7). For each gene, silencing was confirmed by quantitative RT-PCR and was shown to have no detectable influence on phloem sap ingestion by quantifying sugar content in honeydew (S3 Fig). The small GTPase Rab5 can interact with CORVET tethering complexes and regulate the early endocytic pathway by mediating the fusion of early endosomes with endocytic and Golgi-derived vesicles [48, 49]. Rab7, Mon1 and the HOPS complex are responsible for endosomal maturation and membrane fusion at late endosomes and lysosomes [45, 50, 51]. The small GTPase Rab11 controls the traffic of recycling endosomes, which are responsible for delivering cargoes to the plasma membrane in a slow recycling route [52]. These results indicate that the early steps of endosomal trafficking play important roles in virus transport.

**Fig 8:**
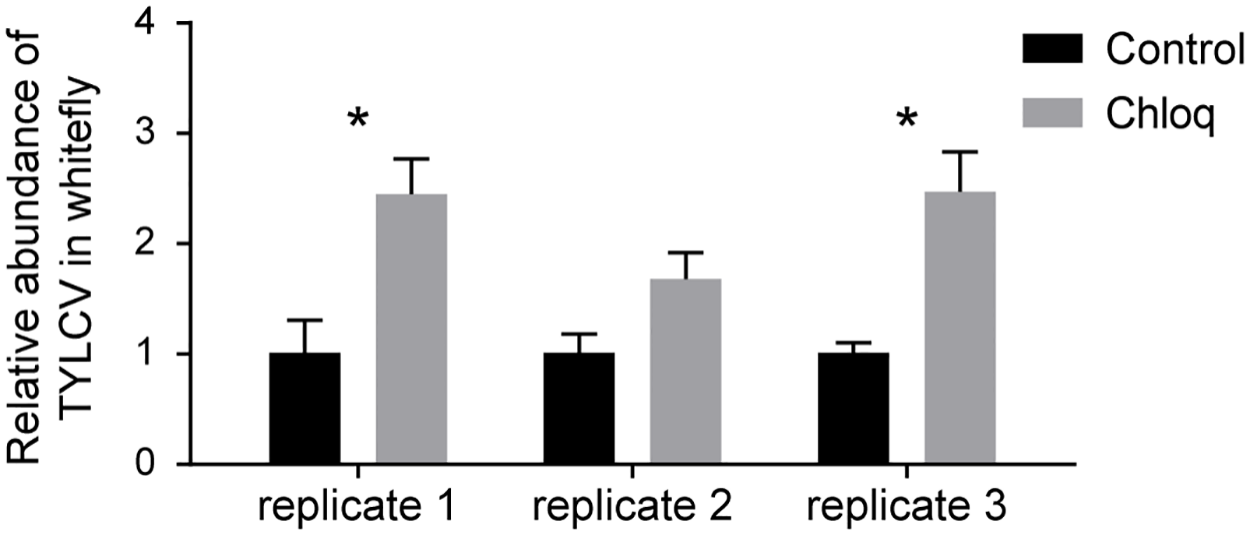
Inhibiting endosomal acidification increases TYLCV content in whitefly. Endosomal acidification was suppressed by feeding whiteflies with Chloq. Then, the whiteflies were allowed to feed on TYLCV-infected plants for 48 h. The abundance of virus in whiteflies was measured by quantitative PCR and compared with that in solvent-fed controls (n = 4). Data shown are mean ± SE. Statistically significant differences are indicated as: *, P<0.05; **, P<0.01.

The maturation of early endosomes into late endosomes is coupled with acidification, which is essential for hydrolytic activity, membrane trafficking, and cargo sorting [53–55]. The exposure of viral particles to low pH or hydrolytic activity in endo-lysosomal system can trigger conformational changes in the structural proteins of viruses, which is a necessary step in some viral life cycles [18, 56]. In order to study whether endosomal maturation and acidification participate in the trafficking process of TYLCV, we used chloroquine (Chloq) to inhibit acid flux and prevent the acidification of endo/lysosome. Rather than inhibiting viral transport, feeding whiteflies with Chloq resulted in a significant increase in virus abundance in two of three independent experiments (Fig 8). This result indicates that instead of aiding the transmission of TYLCV, the maturation and acidification of the endo-lysosomal system caused the proteolytic degradation of viral particles.

### Ultrastructure of HPA-labeled vesicles

**Fig 9:**
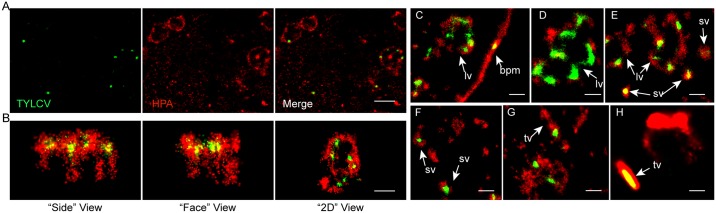
Super-resolution imaging of HPA-labeled vesicles and TYLCV in midgut. Midguts of whiteflies exposed to TYLCV-infected tomato plants for a 3 d AAP were dissected and prepared for super-resolution imaging. (A) STORM images of HPA-labeled vesicles and TYLCV. Lectin HPA formed membrane-like structures and encapsulated TYLCV viral particles in it. Scale bar, 2 μm. (B) 3D STORM image of an HPA-labeled vesicle, allowing the visualization of the vesicle from different angles. The “side” views are from the X axis and the “face” views are from the Y axis. Scale bar, 0.5 μm. (C) Viral particles that have reached the basal plasma membrane (*bpm*) of epithelial cells. Scale bar, 0.5 μm. (D-F) HPA-labeled vesicles with sizes from 200 nm to 2 μm are visible. TYLCV virions can be detected in large vesicles (*lv*) with high pleomorphism and small vesicles (*sv*) that are always spherical. Scale bar, 0.5 μm. (G-H) Tubular vesicles (*tv*) containing TYLCV. Scale bar, 0.5 μm.

To further investigate the architecture of the virus-containing vesicles, we used a single-molecule imaging method, stochastic optical reconstruction microscopy (STORM), to visualize the ultrastructure of these vesicles and their relationship with TYLCV. In STORM images, the empty circular or oval-shaped structures labeled by HPA lectin are speculated to be the membranes of vesicles. The STORM images clearly showed that TYLCV is encapsulated by these HPA-labeled structures (Fig 9A). Three-dimensional STORM images further showed that the vesicles form an ellipsoid-like structure surrounding viral particles (Fig 9B). A full 3D visualization of the HPA-labeled vesicle is provided in S1 Movie.

We subsequently studied the morphologic variability of the HPA-labeled vesicles. In total, three kinds of vesicles were found: large vesicles with diameters ranging from 0.5 to 2 μm, which show great pleomorphism (Fig 9C–9E); small vesicles with diameters of 200 nm and precisely spherical shapes (Fig 9E and 9F); and vesicles with a tubular shape, which might be part of the tubular endosomal network (Fig 9G and 9H). Viral particles were associated with all three types of vesicles as well as with the basal plasma membranes of midgut cells (Fig 9C). These results further confirmed that TYLCV virus particles are transported within vesicles in the cells of the whitefly midgut. Interestingly, most viral particles were localized close to the perimeters of vesicles (Fig 9A–9E), indicating that viruses were recruited to vesicle membranes, likely by receptor-ligand interactions, rather than suspended in the fluid within vesicles. The tubular vesicles may be responsible for sorting virus to the plasma membrane or other destinations [57, 58].

### Sorting nexin (Snx) protein might aid in the secretion of TYLCV

**Fig 10:**
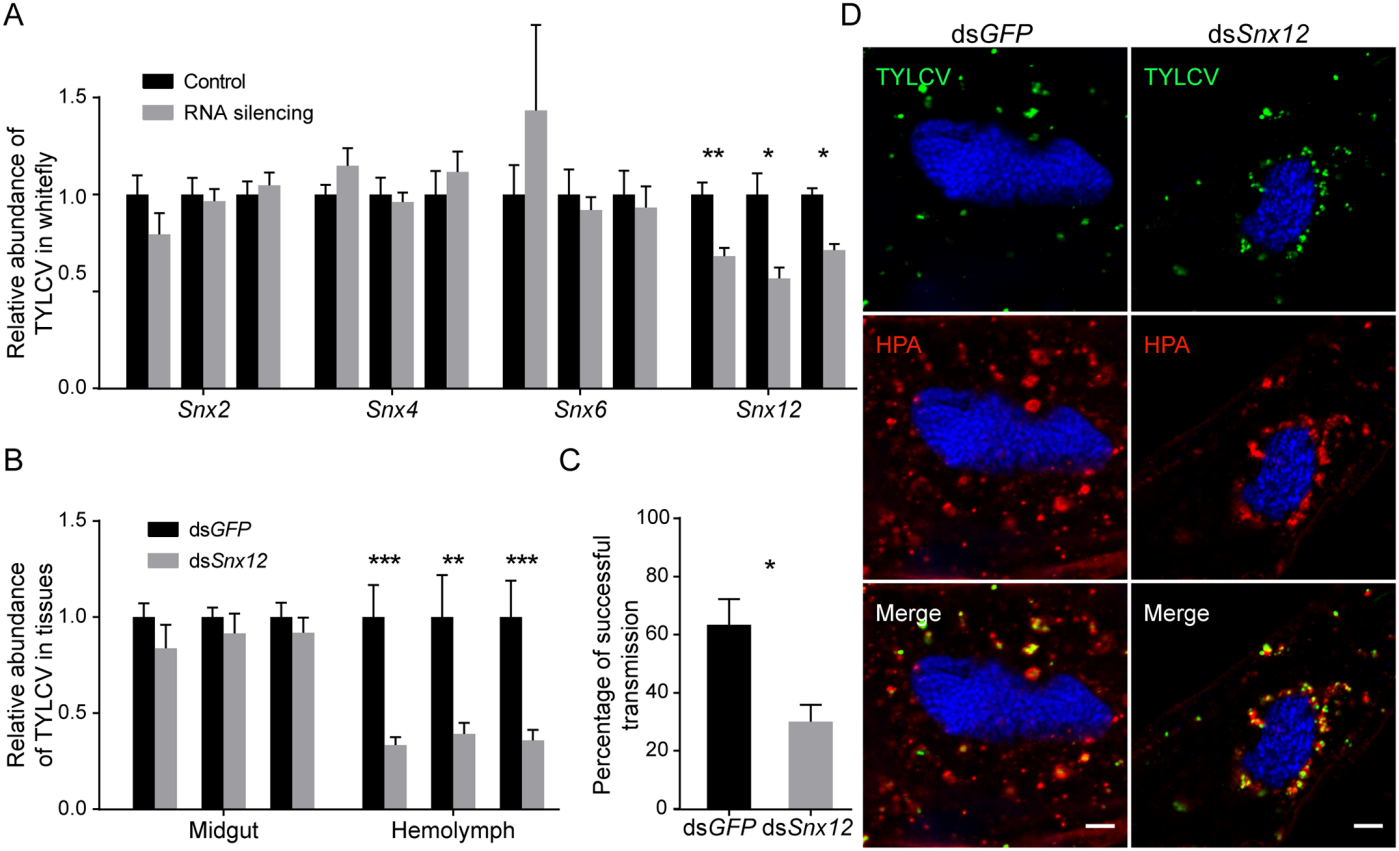
Silencing sorting nexins inhibits TYLCV trafficking. (A-B) Gene silencing of sorting nexins inhibited TYLCV acquisition. After gene silencing by dsRNA injection, whiteflies were transferred to TYLCV-infected plants for virus acquisition. The abundance of virus in the whitefly whole body (A) and in the midgut and hemolymph (B) were measured after 48 h by quantitative PCR and compared with that in controls injected with dsRNA of GFP (for the whitefly whole body n = 4–8; for midgut n = 4; for hemolymph, n = 10–24). (C) Silencing sorting nexins inhibited TYLCV transmission. After dsRNA silencing and 12 h virus acquisition, whiteflies were allowed to feed on tomato seedlings for 48 h and then removed. Percentages of tomato plants infected by the virus were calculated (n = 30). (D) Silencing sorting nexins led to perinuclear distribution of HPA-labeled vesicles. Three days after dsRNA injection, whiteflies were allowed to feed on TYLCV-infected plants for three days and prepared for immunostaining. Colocalization between TYLCV and HPA in whiteflies injected with dsRNA of Snx12 or GFP was quantified as 0.47 ± 0.02 and 0.48 ± 0.03, respectively (Pearson’s coefficient, mean ± SE, n = 14–15). Scale bar, 5 μm. Representative images that were used to generate the data are provided in S10 Fig. Data shown are mean ± SE. Statistically significant differences are indicated as: *, P<0.05; **, P<0.01; ***, P<0.001.

The cargoes in endosomes are sorted depending on tubular-shaped membrane compartments, which allow the packaging of mainly membrane-bound components separately from luminal contents [58]. These tubules are induced and stabilized through a phosphatidylinositol-3-monophosphate (PtdIns3P) binding protein family, Snx [59]. We searched the whitefly genome and identified four Snx genes: Snx2, Snx4, Snx6 and Snx12. Snx2 and Snx6, which form a membrane-deforming subcomplex of retromer complex, are involved in retromer-mediated retrieval transport from endosomes to the Golgi apparatus; Snx4 can regulate recycling transport of the transferrin receptor to the plasma membrane; and Snx12 is homologous to Snx3, which may be involved in the regulation of endocytosis, endosomal sorting, and signaling [59–61]. These genes were subjected to RNA silencing analysis to test their influence on virus transport. The silencing efficiency was confirmed, and the silencing had no appreciable influence on phloem sap ingestion (S5 Fig). Only the *Snx12*-silenced whiteflies had significantly lower TYLCV viral abundance than control whiteflies after feeding on infected plants, suggesting that Snx12 could be involved in virus transport in whitefly midgut cells (Fig 10A). We further examined the role of Snx12 by quantifying virus abundance in the midgut and hemolymph of whiteflies. Silencing of *Snx12* caused a non-significant decrease in the quantity of virus in the midgut but significantly reduced virus abundance in hemolymph (Fig 10B), indicating that Snx12 may play a role in the secretion of virus into hemolymph. The ability of whiteflies to transmit virus to new plants was also tested post-*Snx12* silencing. Notably, silencing of *Snx12* depressed the transmission of TYLCV by whiteflies (Fig 10C). Immunofluorescence analysis further showed that virus-containing vesicles were localized to the perinuclear region after silencing of *Snx12* (Fig 10D). These results further validat the importance of endosomes in TYLCV transport.

### Conservation of an endosomal trafficking route in begomoviruses

**Fig 11:**
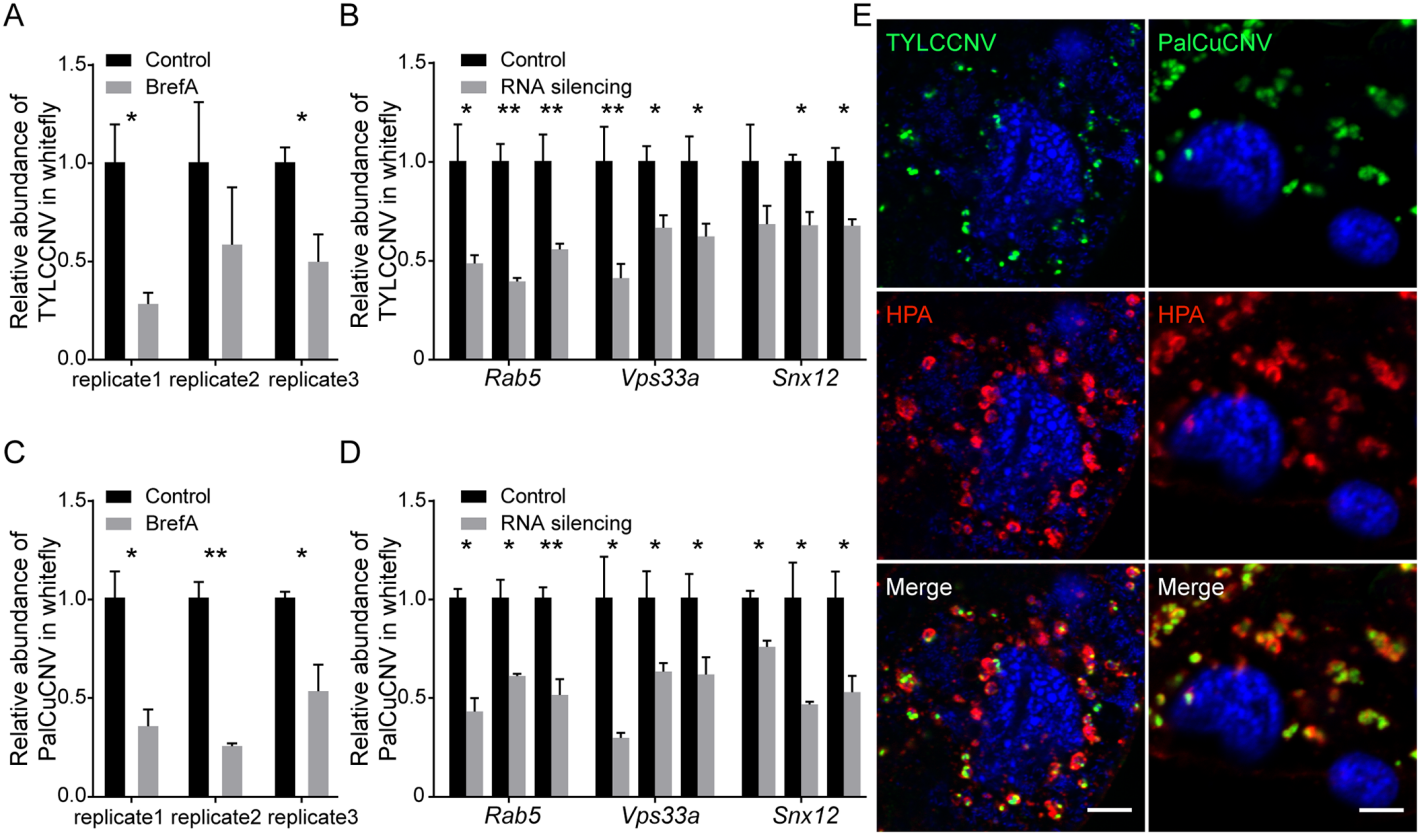
Endosomal trafficking route is conserved among begomoviruses. (A, C) Feeding with BrefA inhibited TYLCCNV and PalCuCNV acquisition. Whiteflies were fed with BrefA to disrupt vesicle trafficking and transferred to TYLCCNV- or PalCuCNV-infected tomato plants for virus acquisition. The abundance of virus in the whiteflies was measured after 48 h by quantitative PCR and compared with that in ethanol-fed controls (n = 4). (B, D) Silencing endosomal genes inhibited the acquisition of both viruses. Whiteflies were injected with dsRNA of target genes and transferred to TYLCCNV- or PalCuCNV-infected plants. The abundance of virus in the whiteflies was quantified 48 h later and compared with that in controls injected with dsRNA of GFP (n = 4–9). (E) Subcellular localization of TYLCCNV and PalCuCNV in midgut cells. Midguts of whiteflies exposed to TYLCCNV- or PalCuCNV-infected plants for a 3 d AAP were dissected and prepared for immunofluorescence. Coat proteins of TYLCCNV and PaLCuCNV were identified using specific antibodies. Colocalization between HPA and TYLCCNV or PalCuCNV was quantified as 0.46 ± 0.03 and 0.41 ± 0.06, respectively (Pearson’s coefficient, mean ± SE, n = 9–12). Scale bar, 5 μm. Representative images that were used to generate the data are provided in S11 Fig. Data shown are mean ± SE. Statistically significant differences are indicated as: *, P<0.05; **, P<0.01.

Finally, we tested the role of endosomes in the transmission of two additional begomoviruses closely related to TYLCV, *Papaya leaf curl China virus* (PalCuCNV) and *Tomato yellow leaf curl China virus* (TYLCCNV) [62, 63]. Whiteflies were fed with inhibitor or injected with dsRNA as described in previous sections. Feeding whiteflies with BrefA significantly inhibited the acquisition of these viruses by whiteflies in five of six independent experiments (Fig 11A and 11C). Silencing of *Rab5*, *Vps33a* and *Snx12*, which are responsible for early steps of endosomal trafficking, significantly reduced the abundance of both viruses in whitefly (Fig 11B and 11D). We further observed the distribution of these two begomoviruses in the midgut of whitefly by immunofluorescence microscopy. Both viruses were colocalized nicely with HPA-labeled vesicles (Fig 11E). These results suggest that early endosomes are a common route for the transport of begomoviruses in their insect vectors.

## Discussion

The transport of virus from the gut lumen into the hemolymph of the insect vector is an important step in the circulative transmission of plant viruses [13, 14]. Brault and colleagues proposed that plant luteoviruses, which undergo circulative transmission, cross the aphid gut epithelium through a transcytosis process dependent on clathrin-mediated endocytosis [64]. However, the detailed trafficking processes for plant viruses in their insect vectors are poorly understood. Our experiments in the present study demonstrated that vesicle trafficking plays important roles in TYLCV intracellular transport. We showed that TYLCV accumulates in HPA-labeled structures. Silencing the CORVET complex and Rab5 can inhibit virus acquisition, suggesting that the early steps of endosomal trafficking play important roles in virus transport. In addition, the transport of TYLCV is probably independent of the Golgi apparatus, late endosomes and recycling endosomes. These results suggest that TYLCV might be transported directly from early endosomes to the basal plasma membrane in a fast recycling route. Nevertheless, the present data cannot rule out the participation of other non-classical pathways. For example, Nonnenmacher *et al*. [20] showed that AAV can be transported to the Golgi apparatus through a non-canonical retrograde transport pathway that is independent of the retromer complex. Thus, TYLCV may be transported to the Golgi apparatus through an unknown pathway and then rapidly secreted to the hemolymph once it arrives.

Many viruses need to be delivered between several kinds of vesicles during their infection process. However, we showed that the transmission of TYLCV was independent of the Golgi apparatus, late endosomes, lysosomes, and recycling endosomes. This suggests that the trafficking of TYLCV in vector cells might be a simpler process than the infection processes characteristic of pathogenic viruses. Pathogenic viruses need to take advantage of specific proteins or environmental conditions in vesicles to become infectious and release their hereditary material into the cytoplasm of host cells [17–19]. For example, Ebola virus particles need to be transported from endosomes to lysosomes before their cytoplasmic release [21]. After internalization, SV40 accumulates in the smooth ER as part of its productive infectious route [65]. The lysosomal cholesterol transporter protein Niemann–Pick C1 and the misfolded protein response machinery in the ER are thought to aid the escapes of Ebola virus and SV40, respectively, from their vesicular compartments [21, 66]. However, TYLCV and other vectored plant viruses that undergo circulative transmission have a different destination: transport into the hemolymph, followed by secretion from the salivary gland into host plants. Therefore, the delivery of virus from early endosomes to other vesicles might be unnecessary.

Ultrastructural analysis of the virus-containing vesicles showed three types of vesicles that differ in the size and shape. The large vesicles may represent endosomes in which TYLCV has accumulated, and the small vesicles with spherical or tubular shapes might represent a previously described group of vesicles that bud from a donor compartment and are responsible for shuttling transport [57]. Since the basal plasma membrane but not the apical membrane of epithelial cells was labeled by lectin HPA, we hypothesize that shuttling vesicles bud from TYLCV-containing vesicles and are targeted to the basal plasma membrane or to other HPA-labeled vesicles. Interestingly, most viral particles were found near or connected to the membrane rather than being suspended in the lumen of vesicles. This indicates that TYLCV might be recruited to the membrane by specific receptor-ligand interactions and that this interaction could be important in its intracellular transport. It will be fascinating to clarify whether the same receptor is responsible for internalization and for intracellular transport. If so, this will raise the question of why and how this receptor is transported from the apical to the basal membrane of epithelial cells. If different receptors are involved, what mechanism promotes the disassociation of viral particles from the first receptor and its association with the second?

Our results also showed that inhibiting endosomal acidification leads to higher TYLCV content in whiteflies, suggesting that the virus may be transported to and degraded in lysosomes. However, no TYLCV particles were observed within late endosomes, and disrupting late endosomes or lysosomes by RNA silencing had no influence on virus content. In recent years, many studies have demonstrated that acidic degradation of extracellular cargo is not limited to lysosomes but can occur further upstream in the endo-lysosomal system [67], suggesting that TYLCV could be degraded in early endosomes. However, because we did not have access to antibodies against lysosomes, it is still unclear which of these possibilities is correct.

**Fig 12:**
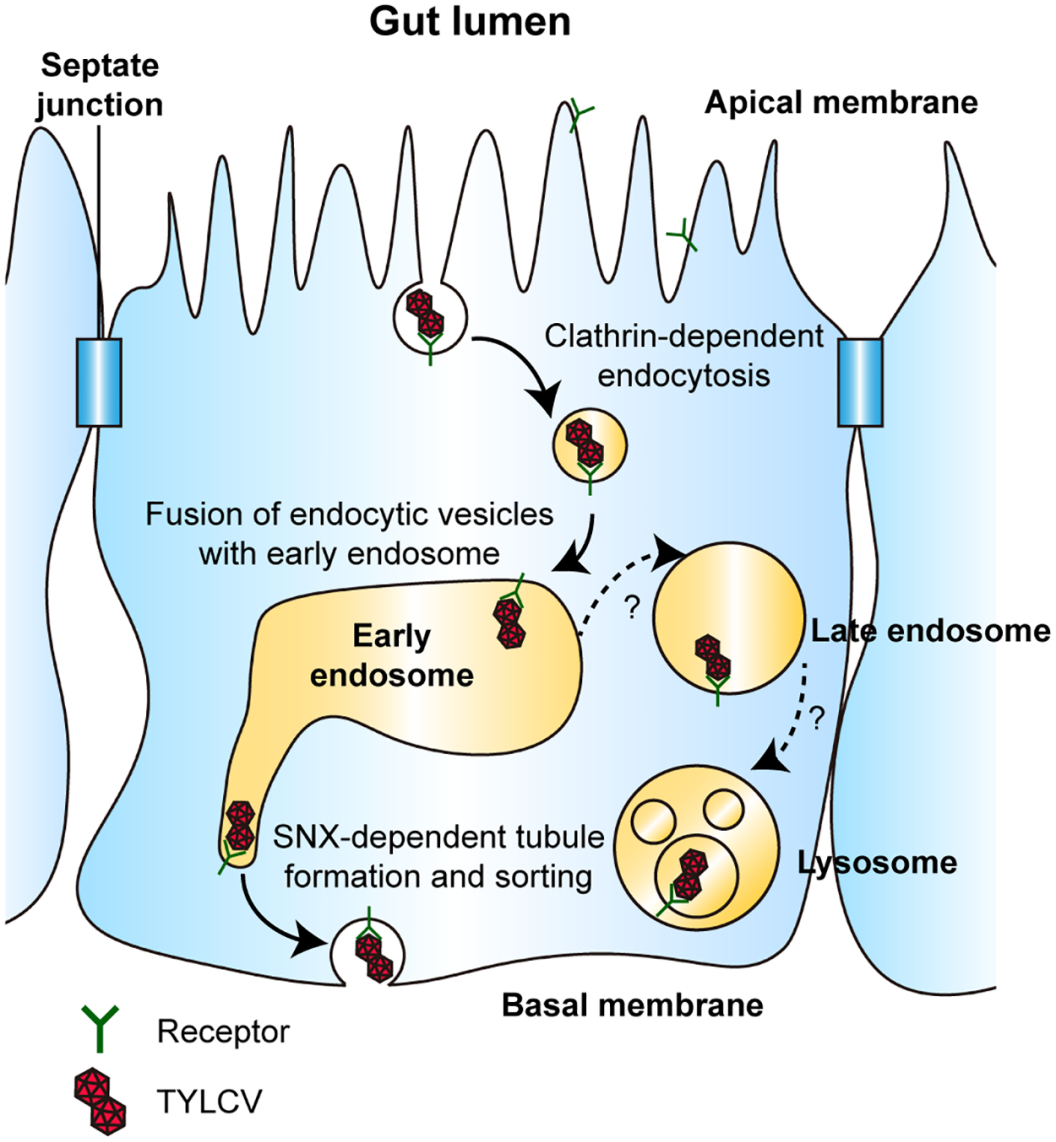
Model of begomovirus trafficking in epithelial cells of whitefly midgut. Following clathrin-mediated endocytosis, begomoviruses are first delivered to early endosomes in a process mediated by Rab5 and the CORVET complex. Next, some viruses are transported directly to the basal membrane of epithelial cells in a process dependent on tubular vesicles induced by Snx12. Inhibiting endosomal acidification leads to increased virus abundance in whitefly, indicating that acid hydrolases in the endo-lysosomal system are responsible for virus degradation. However, whether begomoviruses are transported to late endosomes or lysosomes remains uncertain.

Taken together, our results indicate that early steps of endosomal trafficking play important roles in begomovirus transport. Based on the present experiments and the studies of others [27, 28, 64, 68, 69], we conclude that begomoviruses are first delivered to early endosomes after clathrin-mediated endocytosis, then transported directly to the basal membrane of midgut epithelial cells. The fusion between virus-containing endocytic vesicles and early endosomes is mediated by Rab5 and the CORVET complex. Then, some of the viral particles may be transported to the basal membrane by tubular vesicles induced by Snx12 (Fig 12). Whether some viral particles are transported to lysosomes remains uncertain.

To our knowledge, this is the first study regarding the intracellular trafficking of begomoviruses within their insect vectors. The exact transport pathway of begomoviruses in epithelial cells may be more complicated than the proposed model because these cells are polarized, with apical and basal domains [53]. For example, sorting to the apical and basal membranes are governed by different mechanisms and carried out by different vesicles [70]. Intriguingly, this virus is endocytosed on the apical membrane and exocytosed on the basal membrane. Observing virus transport in live cells may help future researchers better understand the process.

## Methods

### Insects, viruses, and plants

Middle East-Asia Minor 1 whiteflies (MEAM1, previously referred as the ‘B biotype’) were collected from Zhejiang province, China and maintained in the laboratory. Whiteflies were reared on cotton (*Gossypium hirsutum* cv. Zhe-Mian 1793) plants in climate chambers at 26 ± 1°C with a photoperiod of 14 h/10 h and 70 ± 10% relative humidity. Every three months, the purity of the cultures was monitored by PCR-restriction fragment-length polymorphism analysis and further confirmed by sequencing of cytochrome oxidase I (mtCOI, GenBank accession no. KM821540) as previously reported [71].

Infectious clones of TYLCV, TYLCCNV and PaLCuCNV were kindly provided by Professor Xue-Ping Zhou from the Institute of Biotechnology, Zhejiang University. Briefly, 1.4 copies of the genome of each begomovirus was cloned into a plant transformation vector and transfected into *Agrobacterium tumefaciens*. Tomato (*Solanum lycopersicum* cv. Hezuo 903) plants with 3–4 true leaves were inoculated with an infectious clone of TYLCV, TYLCCNV, or PaLCuCNV to obtain virus-infected plants. They were then cultivated to the 6–7 true leaf stage for further experiments.

### Chemicals

Brefeldin A (BrefA, Enzo Life Sciences) was dissolved in ethanol (Sigma) to make a 5 mM stock solution. Chloroquine phosphate (Chloq, Sigma) was dissolved in sterile water to make a 700 mM stock solution. A stock solution of 50 mM Golgicide A (GolA, Selleck) was prepared in dimethyl sulfoxide (DMSO, Sigma). All stock solutions were diluted 1:1000 in water with 30% sucrose for feeding of whiteflies. For chemical treatment, newly emerged whiteflies were collected and fed through a parafilm membrane for 24 h. In different experiments, 30% sucrose solutions containing 0.1% ethanol, DMSO or water were included as controls.

### Double-stranded RNA (dsRNA) synthesis and microinjection

DNA templates were generated by PCR using primers that contained the T7 promoter at both ends. Then, DNA templates and the MEGAscript T7 Transcription Kit (Ambion, USA) were used to synthesize dsRNA according to the manufacturer’s instructions. DsRNA was subsequently purified using phenol:chloroform extraction and isopropanol precipitation, then resuspended in nuclease-free water. The size and quality of the dsRNA were confirmed by 1% agarose gel electrophoresis, and its quantity was measured using Nanodrop (Thermo Scientific, USA). Primers used for DNA template synthesis are listed in S1 Table.

For RNA silencing, approximately 6 nl of purified dsRNA (8 μg/μl) was injected into the thorax of each adult female whitefly using capillary and FemtoJet (Eppendorf, Germany). For each gene, 200 female whiteflies were injected and dsRNA corresponding to green fluorescent protein (GFP) was included as control. After injection, whiteflies were kept on cotton plants for three days for recovery. Efficiency of dsRNA mediated gene silencing was verified by qRT-PCR.

### Acquisition of virus by whiteflies

After inhibitor feeding or dsRNA injection, about 50 female whiteflies of each treatment group were caged on one leaf, while about 50 control whiteflies were caged on a symmetrical leave of the same virus-infected tomato plant. After 48 h AAP, these whiteflies were collected and a group of 10 female whiteflies was prepared as one sample. For each experiment, 4–6 samples were usually prepared for analysis, and the results are shown as one bar in the graphs (n in each figure represents the number of these samples). The collected whiteflies were homogenized in 200 μl lysis buffer (10 mM Tris-HCl pH 8.4, 50 mM KCl, 0.45% Tween-20, 0.45% Nonidet P-40, 0.2% gelatin and 60 mg/l proteinase K) then incubated at 56°C for 1 h and 100°C for 10 min. The supernatant was used in quantitative PCR (qPCR) after centrifuging at 12,000 rpm for 3 min. For each inhibitor or gene silencing treatment, the experiment was repeated three times.

Quantification of virus titer in midguts and hemolymph were similar. The midguts of female viruliferous whiteflies were dissected in DPBS and washed several times before use. A group of 15 midguts was prepared as one sample. For each experiment, 4 samples were usually prepared for analysis (n in each figure represents the number of samples) and the experiment was repeated three times. Midguts were homogenized in 30 μl lysis buffer and processed in the same way as the whitefly whole bodies. The hemolymph of whitefly was collected from the abdomen of female whiteflies using a capillary with a fine point of ~1 μm in diameter. Hemolymph of each whitefly was homogenized in 10 μl lysis buffer and processed in the same way as the whitefly whole bodies. Hemolymph from two whiteflies was pooled and prepared as one sample. For each experiment, 10–20 samples were usually prepared (n in each figure represents the number of samples), and each experiment was repeated three times.

### Real-time PCR

Quantitative real-time PCR was used to analyze the quantity of virus acquired by the whiteflies and the efficiency of RNAi. Total RNA of whitefly was isolated using TRIzol (Ambion, USA) and reverse transcribed using PrimeScript RT reagent Kit (TaKaRa, Japan) following the manufacturer’s protocol. Quantitative PCR was performed on CFX Connect Real-Time PCR System (Bio-Rad, USA) using the FastStart Essential DNA Green Master (Roche, Switzerland) and custom-designed specific primers to the genes. Actin was used as an internal reference, and relative abundance of TYLCV or transcripts was calculated by 2^-ΔCt^. Primers used for real-time PCR are listed in S1 Table.

### Sugar in whitefly honeydew

After 48 h feeding on virus-infected tomato plant, the whitefly honeydew was collected by washing the leaves and inner surfaces of the micro-cages with sterile water several times. The collected honeydew was diluted to 1 ml. Glucose dissolved in water was used to produce a standard curve. The anthrone reaction was used to determine the content of sugar in the honeydew solution, as described previously [72]. Briefly, anthrone reagent was prepared by dissolving 0.2 g of anthrone in 100 ml 80% (w/w) H_2_SO_4_. Then, 40 μl of the honeydew solution was mixed with 160 μl anthrone reagent and heated as required in a 100°C water bath. The absorbance at 620 nm was determined using a Varioskan Multimode Microplate Reader (Thermo Scientific, USA).

### Transmission of TYLCV by whiteflies

After being fed with inhibitor or injected with dsRNA, the treatment group and its control group were caged on two symmetrical leaves of the same virus-infected tomato plant for 12 h. The whiteflies were then collected and sexed. Female whiteflies were placed in groups of 4 on a true leaf of an uninfected tomato plant at the 3–4 true-leaf stage for 48 h using a micro-cage. Then, whiteflies were removed from tomato plants, and plants were sprayed with imidacloprid (50 mg/l) to kill eggs. After 30 days, plants were evaluated for TYLCV infection based on symptoms and diagnostic PCR.

### Sample preparation for transmission electron microscopy

Midguts of whiteflies that had had a 7-day AAP on TYLCV-infected tomato plant as well as TYLCV-unexposed control whiteflies were dissected with needles in DPBS and washed three times before use. The midguts were first fixed overnight with 2.5% glutaraldehyde in phosphate buffer (0.1M, pH7.0) and washed three times with phosphate buffer; then post-fixed with 1% OsO_4_ in phosphate buffer for 1.5h, washed, dehydrated in a graded series of ethanol (30%, 50%, 70%, 80%, 90%, 95% and 100%) and embedded in Spurr resin. The specimen was sectioned using a LEICA EM UC7 Ultramicrotome. Sections were stained using uranyl acetate and alkaline lead citrate for 5 to 10 min, respectively, and observed in a Hitachi Model H-7650 TEM.

### Immunostaining

Midguts of whiteflies were dissected freshly and fixed for 1 h with 4% paraformaldehyde (PFA, Thermo Fisher, USA) in phosphate-buffer saline (DPBS; PH = 7.4), then excess PFA was washed off with DPBS. Midguts were then permeabilized using 0.4% Triton X-100 in DPBS for 1 h and blocked using 3% BSA in DPBS for 2 h, followed by incubation with primary antibody (overnight at 4°C). Midguts were subsequently incubated with secondary antibody and fluorescence labeled lectins for 2 h at room temperature, and finally fixed in 3% PFA with 0.05% glutaraldehyde in DPBS for 30 min. Monoclonal antibodies that recognize the coat proteins of TYLCV, TYLCCNV, and PalCuCNV were kindly provided by Professor Jian-Xiang Wu. The polyclonal antibodies against alpha-mannosidase 2 (Man2), Rab5, Rab7 and TGN46 were produced by GenScript using synthetic peptides conjugated to KLH. The other antibodies and conjugated lectins were ERp57 polyclonal antibody (PA5-29810; Invitrogen), Rab11 polyclonal antibody (2413; Cell Signaling Technology), goat anti-mouse IgG Alex 488 (A11029; Invitrogen), goat anti-rabbit IgG Alex 647 (A21245; Invitrogen), goat anti-rabbit IgG Alex 488 (A11034; Invitrogen), lectin GS-II Alex 647 (L32451; Invitrogen), lectin HPA Alex 647 (L32454; Invitrogen), and lectin WGA Alex 647 (W32466; Invitrogen).

For confocal imaging, midguts were mounted in Fluoroshield Mounting Medium with DAPI (Abcam, USA) and viewed under LSM 780 (ZEISS, Germany). ImageJ and JACoP were used for colocalization analysis with default parameters.

For stochastic optical reconstruction microscopy, midguts were first immobilized on the bottom of Glass Bottom Cell Culture Dishes (NEST, China) and immersed in STORM imaging buffer containing cysteamine (MEA) and 2-mercaptoethanol (50 mM Tris-HCl pH = 8.0, 10 mM NaCl, 10% Glucose, 5 mM MEA, 0.5% 2-mercaptoethanol, 560 μg/ml Glucose Oxidase, 34 μg/ml Catalase). Prior to STORM imaging, a strong laser was used to inactivate most fluorophores. STORM acquisition was then started with imaging cycles containing one frame of activation laser illumination (405 nm) followed by eight frames of imaging laser illumination (561 nm, 657 nm). Typically, one STORM image containing more than 50,000 pictures was acquired per hour. These data were subsequently used for analysis of the locations of probes.

### Lectin affinity capture and mass spectrometry

HPA lectin was conjugated to NHS Mag Sepharose magnetic beads (GE Healthcare, USA), following the manufacturer’s protocol. One g of whiteflies was ground in liquid nitrogen and dissolved in TBS (50 mM Tris, 150 mM NaCl, pH 7.5) with 0.4% NP-40. Then, the solution was incubated overnight at 4°C with magnetic beads coupled with lectin HPA and washed three times with TBS. Finally, proteins on the magnetic beads were eluted using elution buffer (0.1 M glycine-HCl, pH 2.5) and subjected to mass spectrometry analysis at Shanghai Applied Protein Technology Co., Ltd. Magnetic beads that were not coupled with lectin HPA were included as a control.

### Statistical analysis

The Mann-Whitney U test was used for comparisons of the relative abundance of virus in whitefly and the expression levels of genes. Goodness-of-fit test for independence was applied to comparisons of the transmission efficiency of TYLCV by whitefly. A P-value < 0.05 was considered as the threshold for significant difference. All the statistical analyses were performed with SPSS 20.0 (SPSS Inc., USA).

## Supporting information

S1 Fig BrefA treatment successfully disrupts Golgi apparatus. Whiteflies that had been fed with BrefA (A) or ethanol control (B) for 2 days were prepared for immunofluorescence. The effect of BrefA treatment on the Golgi apparatus was confirmed by the mislocalization of TGN46.
(TIF)

S2 Fig GolA treatment and silencing of retromer do not affect whitefly feeding. (A) Confirmation of dsRNA-mediated gene silencing. Genes were silenced by injecting dsRNA into hemolymph of whitefly. Gene expression levels were measured by qRT-PCR at 72 h post injection (n = 4–5). (B) The quantity of honeydew excreted by whiteflies after dsRNA injection. (C) The quantity of honeydew excreted by whiteflies after feeding with GolA. The honeydew produced per whitefly per day was quantified based on its sugar content (n = 5–8). The lack of significant effects on honeydew production indicates that neither GolA nor RNA silencing of retromer complex influence whitefly phloem sap feeding. (D) Whiteflies that had been fed with GolA (right) or DMSO control (left) for 2 days were prepared for immunofluorescence. Data shown are mean ± SE.
(TIF)

S3 Fig Silencing of genes involved in endosomal trafficking does not affect whitefly feeding. (A) Confirmation of dsRNA-mediated gene silencing. Genes were silenced by injecting dsRNA into hemolymph of whiteflies. mRNA levels were measured by qRT-PCR at 72 h post injection (n = 4–5). (B) The quantity of honeydew excreted by whiteflies after dsRNA injection. The honeydew produced per whitefly per day was quantified based on its sugar content (n = 5–8). Data shown are mean ± SE.
(TIF)

S4 Fig Chloroquine treatment does not affect whitefly feeding. The quantity of honeydew excreted by whiteflies after feeding with chloroquine (Chloq). The honeydew produced per whitefly per day was quantified based on its sugar content (n = 5–8). Data shown are mean ± SE.
(TIF)

S5 Fig Silencing of sorting nexins does not affect whitefly feeding. (A) Confirmation of dsRNA-mediated gene silencing. Genes were silenced by injecting dsRNA into hemolymph of whiteflies. Gene expression levels were measured by qRT-PCR at 72 h post injection (n = 4–5). (B) The quantity of honeydew excreted by whitefly after dsRNA injection. The honeydew produced per whitefly per day was quantified based on its sugar content (n = 5–8). Data shown are mean ± SE.
(TIF)

S6 Fig Images for localizing TYLCV and lectins. Representative images that were used to generate Pearson’s coefficient in Fig 3. Midguts of whiteflies exposed to TYLCV-infected tomato plants for a 3 d AAP were dissected and prepared for immunofluorescence. (A) Blue signal indicates the cell nucleus. Green signal indicates TYLCV. Red signal indicates labelling of lectins WGA, GS-II or HPA. (B) TYLCV and HPA were localized after treatment with BrefA or ethanol control. Scale bar 5 μm.
(TIF)

S7 Fig Images for localizing TYLCV after different AAP. Representative images that were used to generate Pearson’s coefficient in Fig 4. Whiteflies were allowed to feed on TYLCV-infected plants for different AAP and prepared for immunofluorescence. Blue signal indicates the cell nucleus. Green signal indicates TYLCV. Red signal indicates labelling of HPA lectin. Scale bar 5 μm.
(TIF)

S8 Fig Images for localizing TYLCV, Golgi apparatus and ER. Representative images that were used to generate Pearson’s coefficient in Fig 5. Midguts of whiteflies exposed to TYLCV-infected tomato plants for a 3 d AAP were dissected and prepared for immunofluorescence. (A) Blue signal indicates the cell nucleus. Green signal indicates TYLCV. Red signal indicates Golgi apparatus (Man2) or ER (ERp57). (B) Blue signal indicates cell nuclei. Green signal indicates Golgi apparatus (Man2). Red signal indicates labelling of HPA lectin. Scale bar 5 μm.
(TIF)

S9 Fig Images for localizing TYLCV and endosomes. Representative images that were used to generate Pearson’s coefficient in Fig 6. Midguts of whiteflies exposed to TYLCV-infected tomato plants for a 3 d AAP were dissected and prepared for immunofluorescence. Blue signal indicates the cell nucleus. Green signal indicates TYLCV. Red signal indicates early endosomes (Rab5), late endosomes (Rab7) or recycling endosomes (Rab11). Scale bar 5 μm.
(TIF)

S10 Fig Images for localizing TYLCV and HPA lectin after *SNX12* silencing. Representative images that were used to generate Pearson’s coefficient in Fig 10. Three days after dsRNA injection, whiteflies were allowed to feed on TYLCV-infected plant for three days and prepared for immunostaining. Blue signal indicates the cell nucleus. Green signal indicates TYLCV. Red signal indicates labelling of HPA lectin. Scale bar 5 μm.
(TIF)

S11 Fig Images for localizing PalCuCNV, TYLCCNV and HPA lectin. Representative images that were used to generate Pearson’s coefficient in Fig 11. Midguts of whiteflies exposed to PalCuCNV- or TYLCCNV-infected tomato plants for a 3 d AAP were dissected and prepared for immunofluorescence. Blue signal indicates the cell nucleus. Green signal indicates PalCuCNV or TYLCCN. Red signal indicates labelling of HPA lectin. Scale bar 5 μm.
(TIF)

S1 Movie Three-dimensional view of an HPA-labeled vesicle and TYLCV. (AVI)

S1 Table Information on the primers used in this study. (XLSX)
