# Supplementary figures and images for: Intracellular trafficking of begomoviruses in the midgut cells of their insect vector
Source: PLoS Pathog. 2018 Jan 25;14(1):e1006866. doi: 10.1371/journal.ppat.1006866 (PMC5800681; doi:10.1371/journal.ppat.1006866)

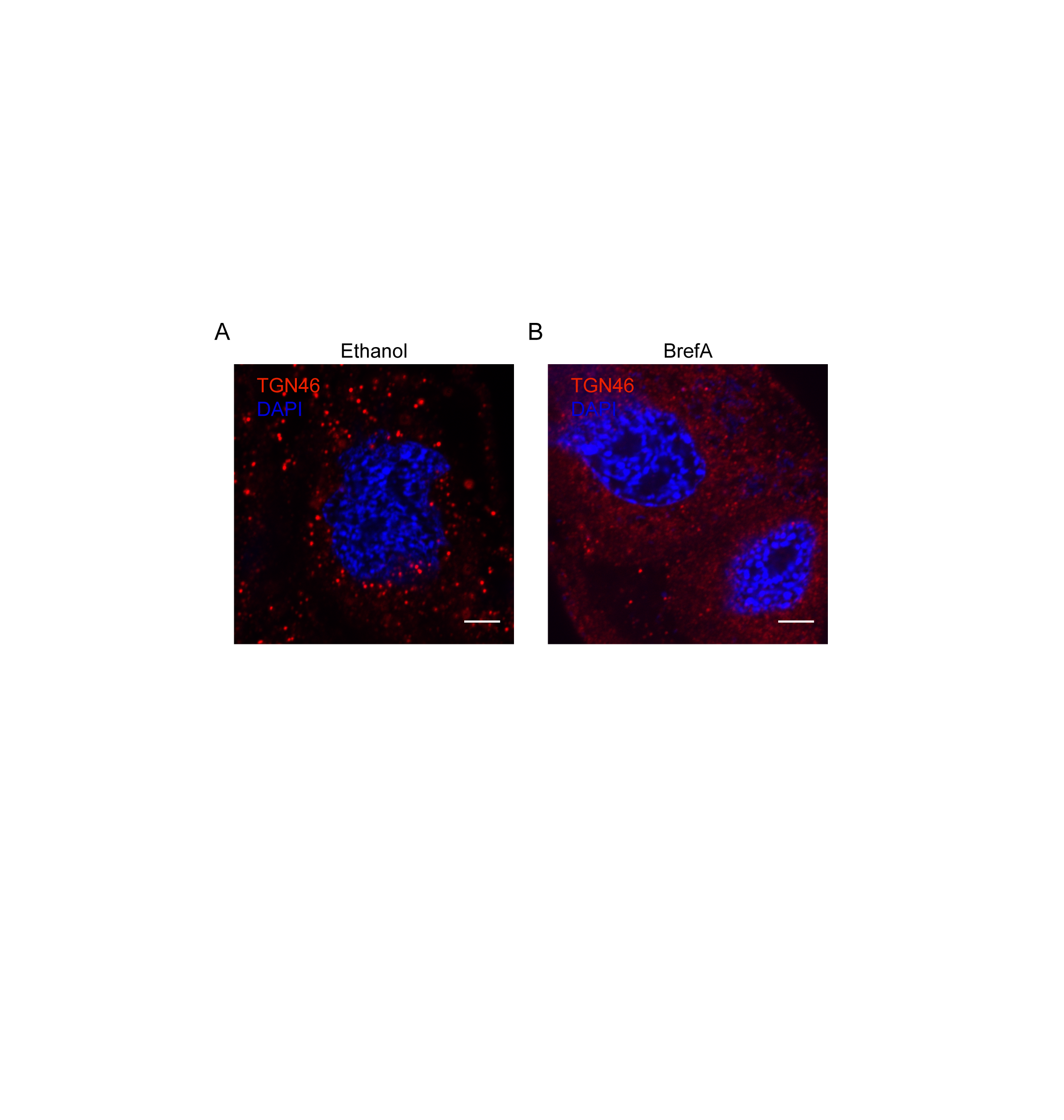

Supplement: S1 Fig — Whiteflies that had been fed with BrefA (A) or ethanol control (B) for 2 days were prepared for immunofluorescence. The effect of BrefA treatment on the Golgi apparatus was confirmed by the mislocalization of TGN46. (TIF) [file ppat.1006866.s001.tif]

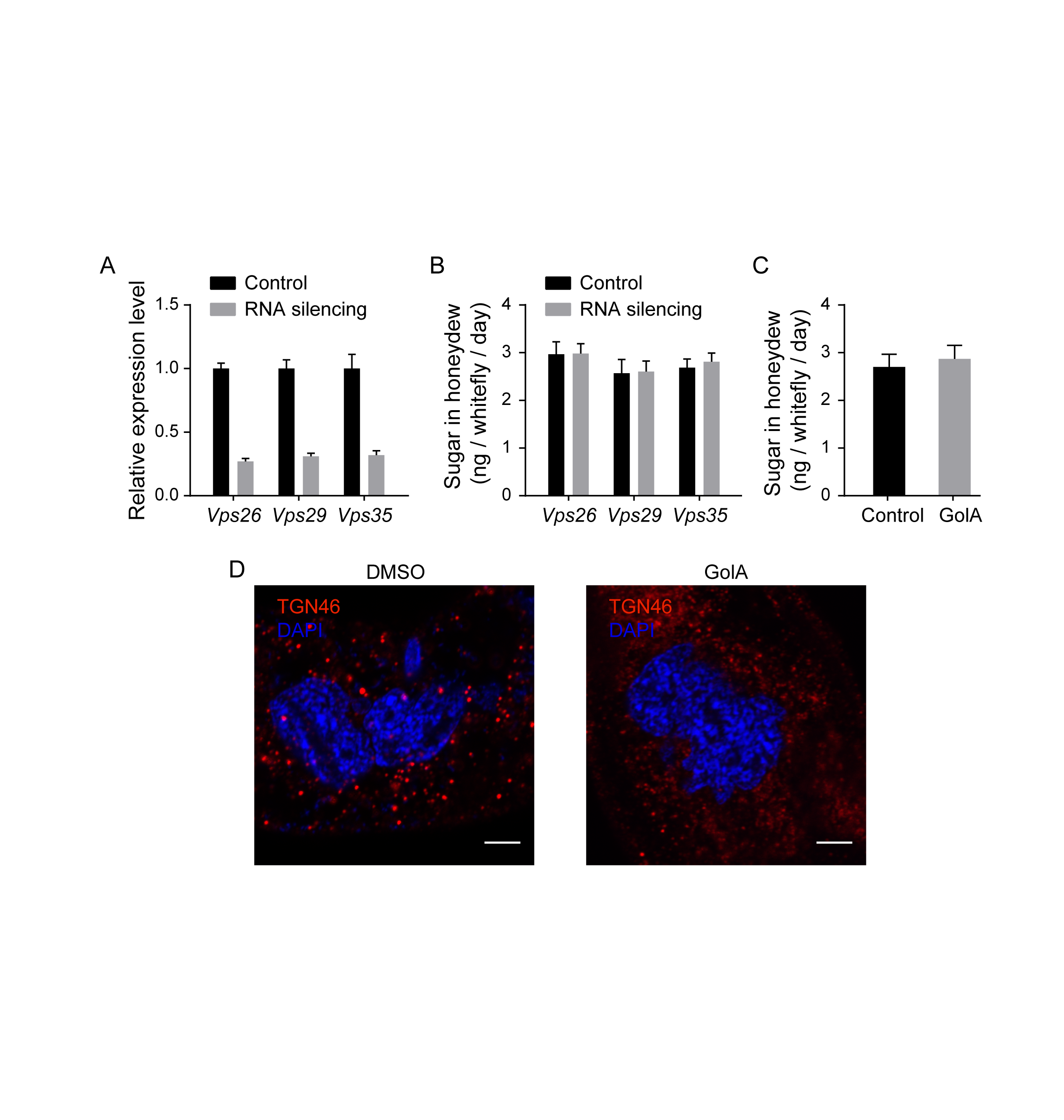

Supplement: S2 Fig — (A) Confirmation of dsRNA-mediated gene silencing. Genes were silenced by injecting dsRNA into hemolymph of whitefly. Gene expression levels were measured by qRT-PCR at 72 h post injection (n = 4–5). (B) The quantity of honeydew excreted by whiteflies after dsRNA injection. (C) The quantity of honeydew excreted by whiteflies after feeding with GolA. The honeydew produced per whitefly per day was quantified based on its sugar content (n = 5–8). The lack of significant effects on honeydew production indicates that neither GolA nor RNA silencing of retromer complex influence whitefly phloem sap feeding. (D) Whiteflies that had been fed with GolA (right) or DMSO control (left) for 2 days were prepared for immunofluorescence. Data shown are mean ± SE. (TIF) [file ppat.1006866.s002.tif]

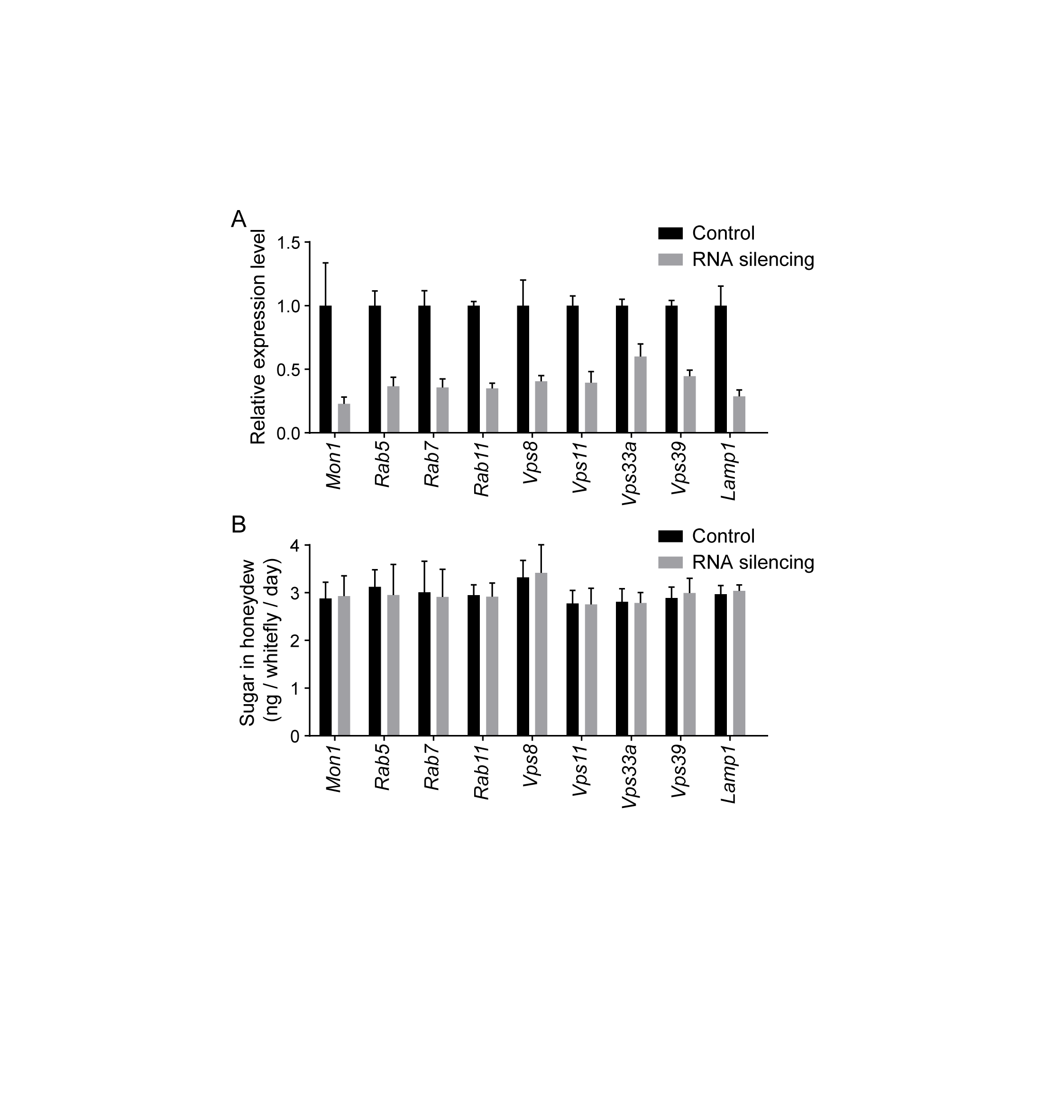

Supplement: S3 Fig — (A) Confirmation of dsRNA-mediated gene silencing. Genes were silenced by injecting dsRNA into hemolymph of whiteflies. mRNA levels were measured by qRT-PCR at 72 h post injection (n = 4–5). (B) The quantity of honeydew excreted by whiteflies after dsRNA injection. The honeydew produced per whitefly per day was quantified based on its sugar content (n = 5–8). Data shown are mean ± SE. (TIF) [file ppat.1006866.s003.tif]

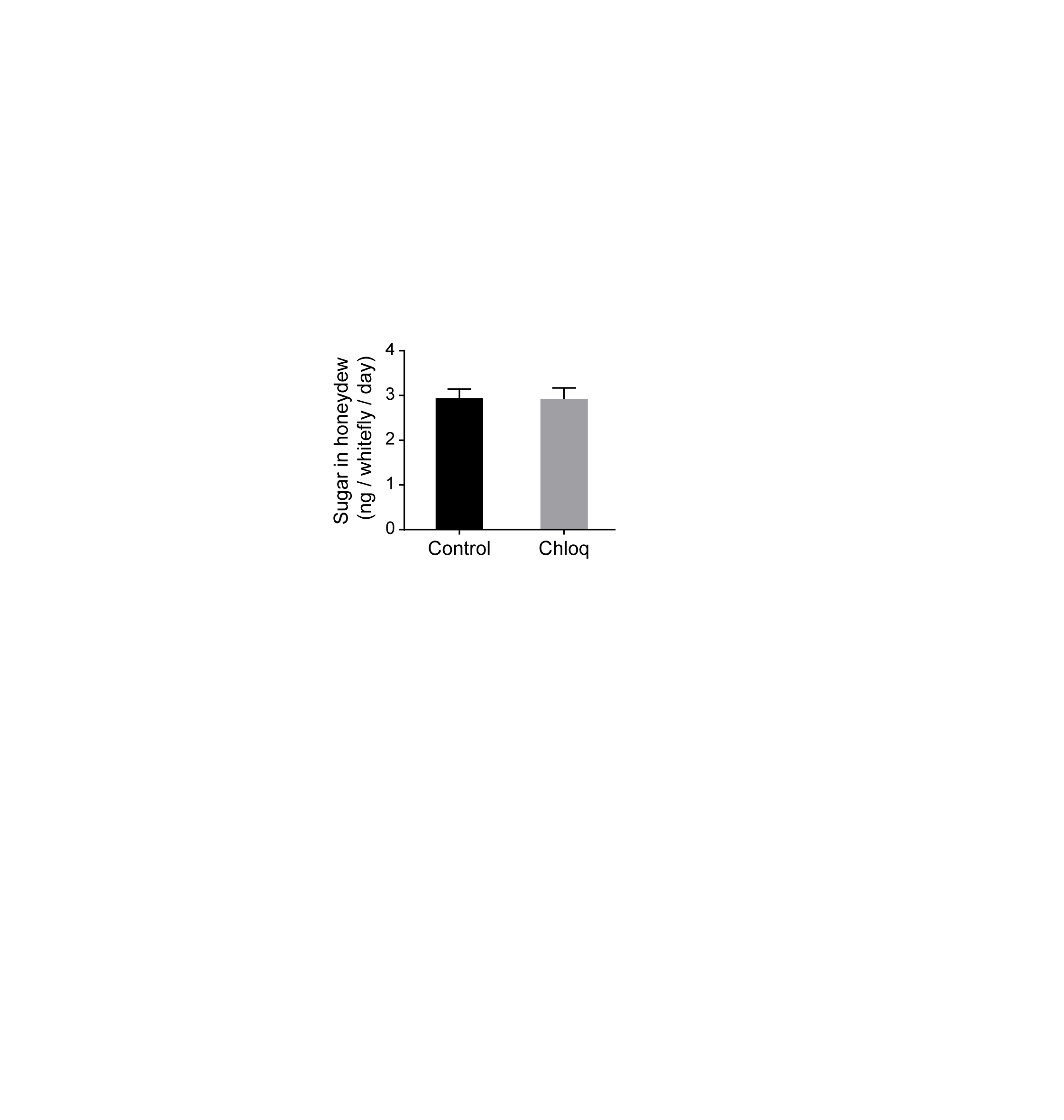

Supplement: S4 Fig — The quantity of honeydew excreted by whiteflies after feeding with chloroquine (Chloq). The honeydew produced per whitefly per day was quantified based on its sugar content (n = 5–8). Data shown are mean ± SE. (TIF) [file ppat.1006866.s004.tif]

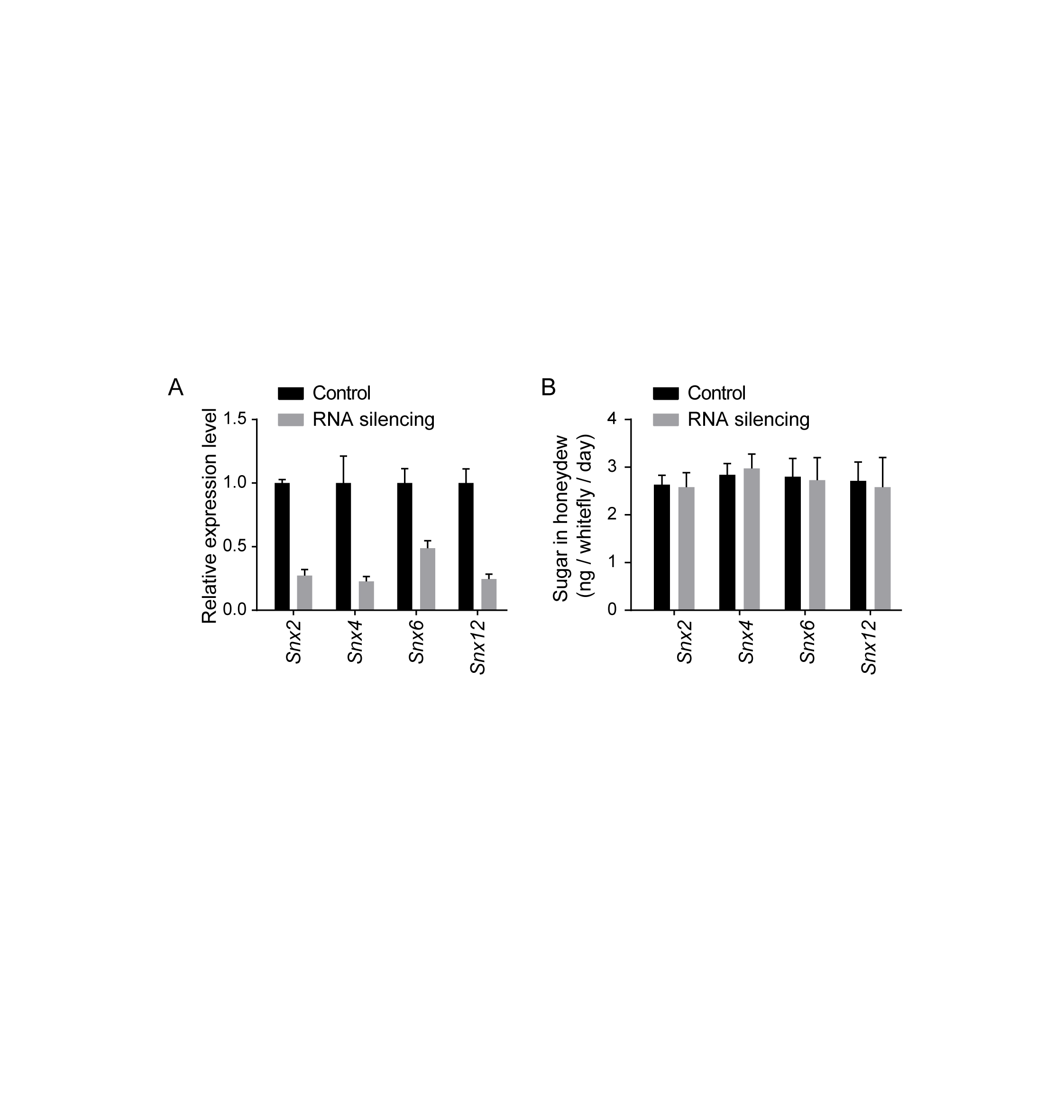

Supplement: S5 Fig — (A) Confirmation of dsRNA-mediated gene silencing. Genes were silenced by injecting dsRNA into hemolymph of whiteflies. Gene expression levels were measured by qRT-PCR at 72 h post injection (n = 4–5). (B) The quantity of honeydew excreted by whitefly after dsRNA injection. The honeydew produced per whitefly per day was quantified based on its sugar content (n = 5–8). Data shown are mean ± SE. (TIF) [file ppat.1006866.s005.tif]

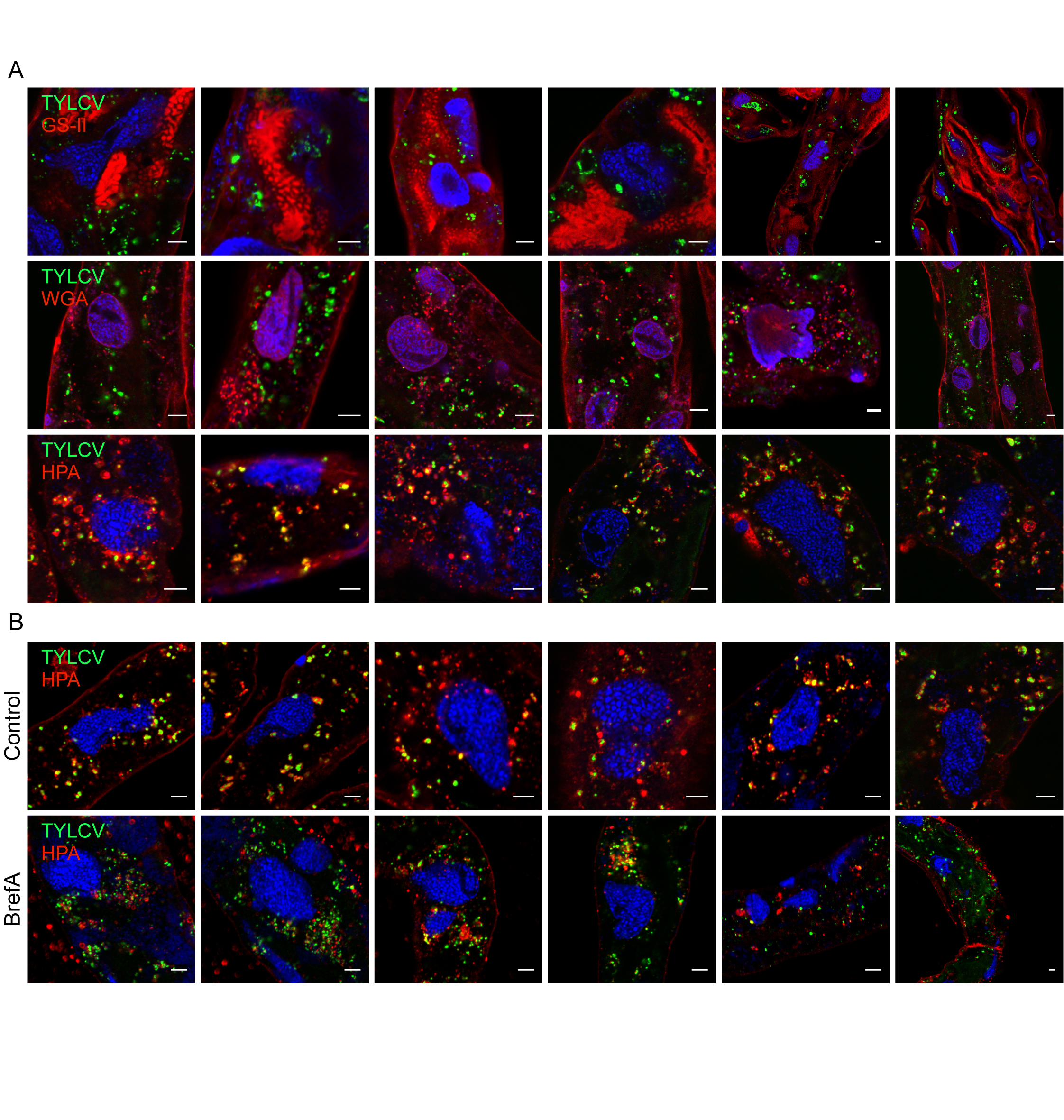

Supplement: S6 Fig — Representative images that were used to generate Pearson’s coefficient in Fig 3. Midguts of whiteflies exposed to TYLCV-infected tomato plants for a 3 d AAP were dissected and prepared for immunofluorescence. (A) Blue signal indicates the cell nucleus. Green signal indicates TYLCV. Red signal indicates labelling of lectins WGA, GS-II or HPA. (B) TYLCV and HPA were localized after treatment with BrefA or ethanol control. Scale bar 5 μm. (TIF) [file ppat.1006866.s006.tif]

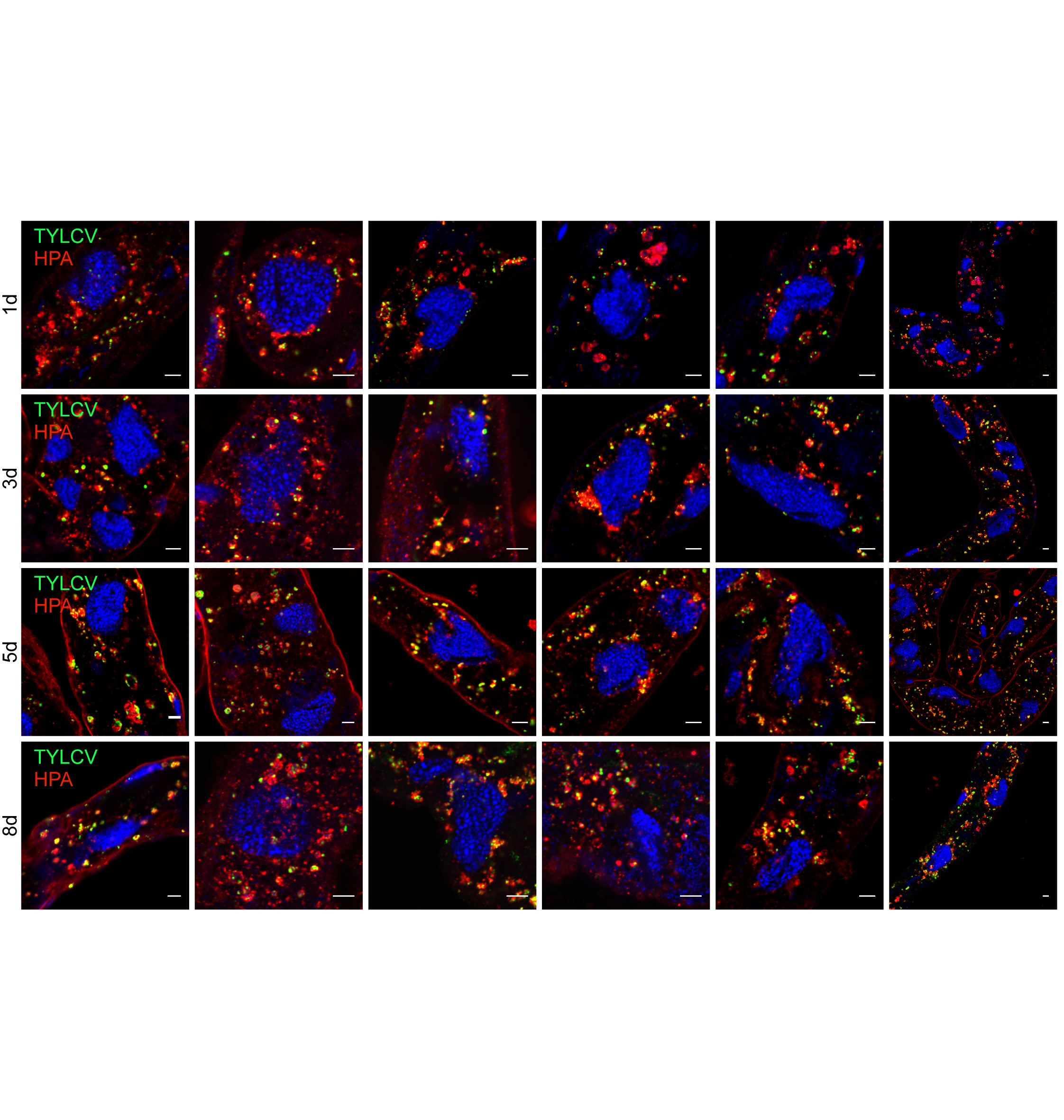

Supplement: S7 Fig — Representative images that were used to generate Pearson’s coefficient in Fig 4. Whiteflies were allowed to feed on TYLCV-infected plants for different AAP and prepared for immunofluorescence. Blue signal indicates the cell nucleus. Green signal indicates TYLCV. Red signal indicates labelling of HPA lectin. Scale bar 5 μm. (TIF) [file ppat.1006866.s007.tif]

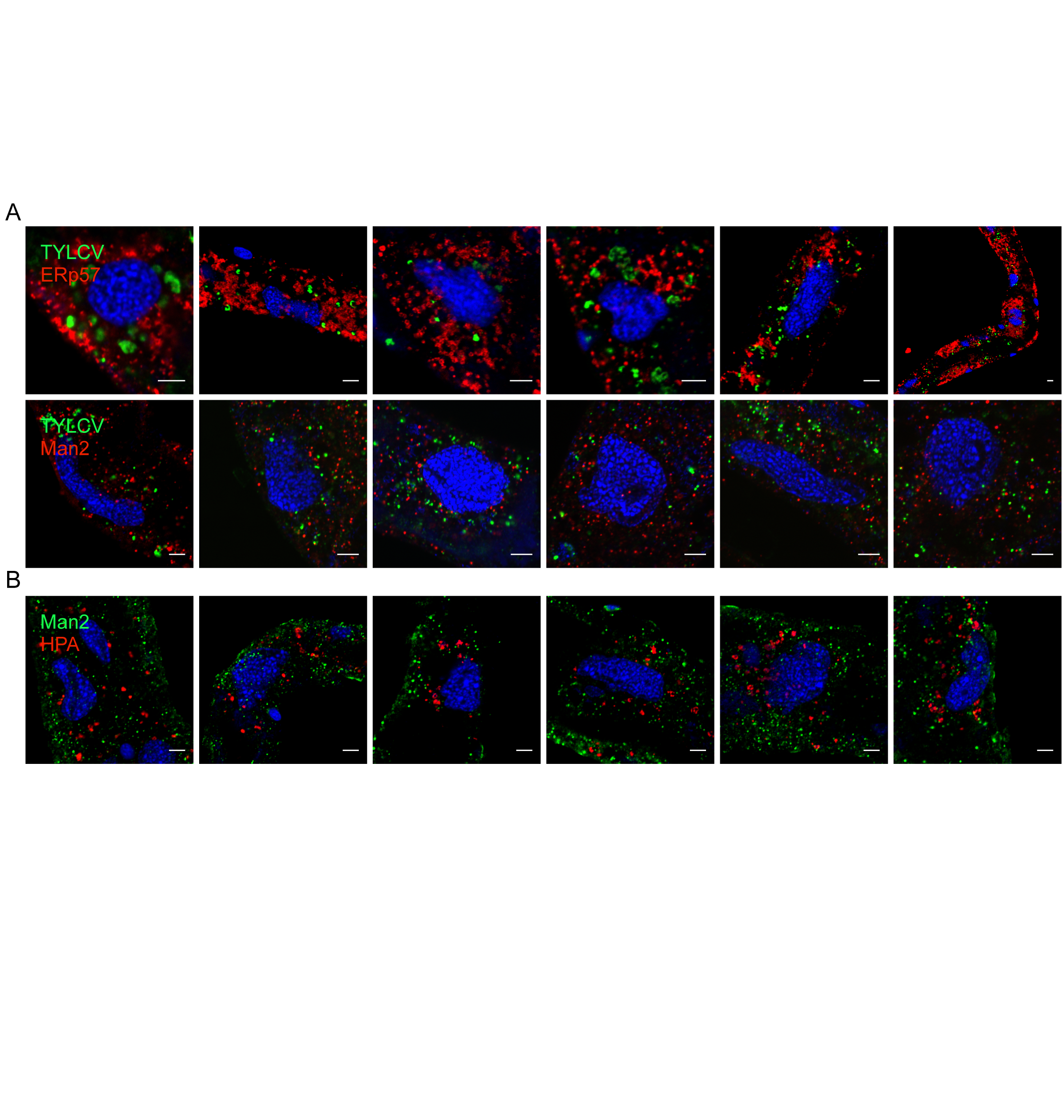

Supplement: S8 Fig — Representative images that were used to generate Pearson’s coefficient in Fig 5. Midguts of whiteflies exposed to TYLCV-infected tomato plants for a 3 d AAP were dissected and prepared for immunofluorescence. (A) Blue signal indicates the cell nucleus. Green signal indicates TYLCV. Red signal indicates Golgi apparatus (Man2) or ER (ERp57). (B) Blue signal indicates cell nuclei. Green signal indicates Golgi apparatus (Man2). Red signal indicates labelling of HPA lectin. Scale bar 5 μm. (TIF) [file ppat.1006866.s008.tif]

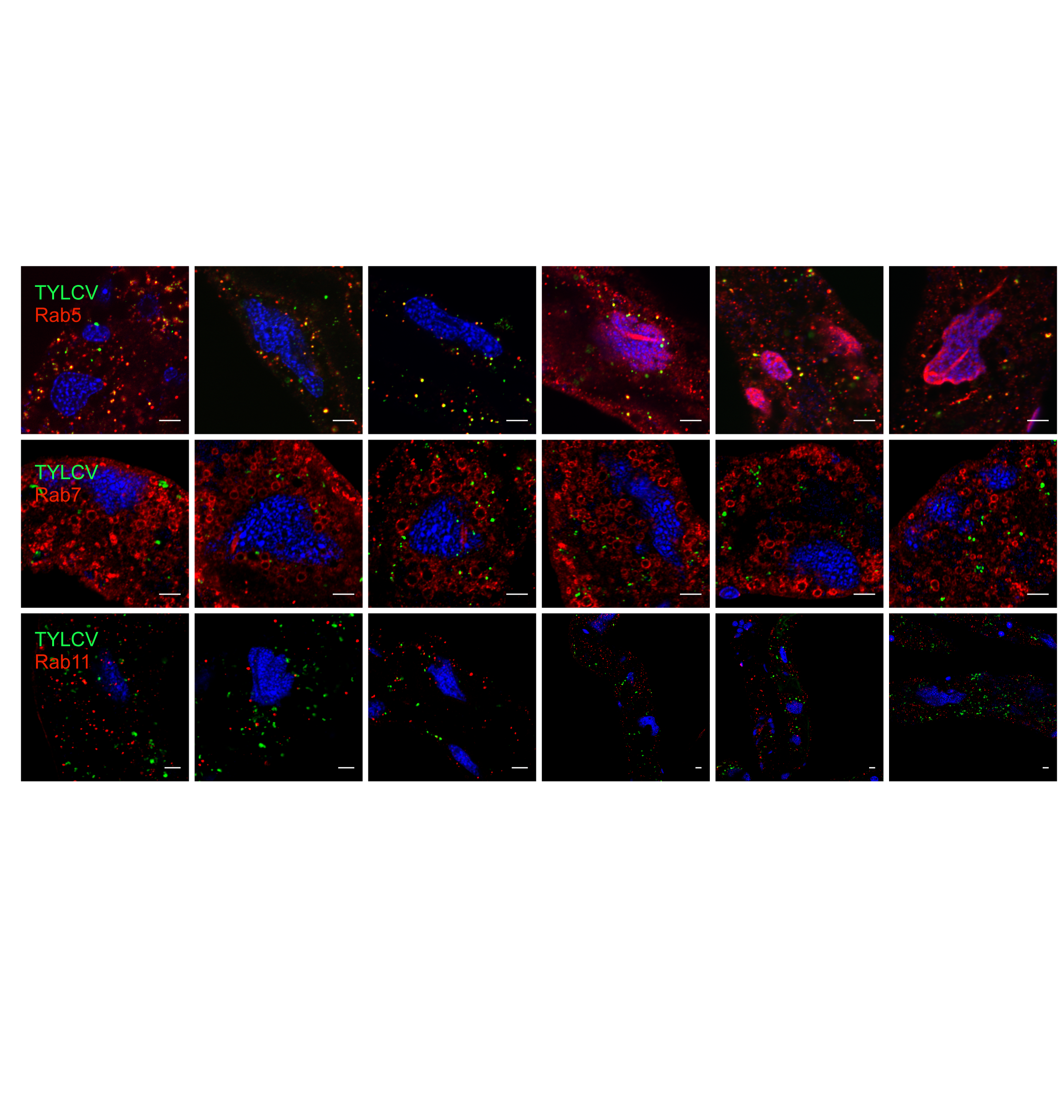

Supplement: S9 Fig — Representative images that were used to generate Pearson’s coefficient in Fig 6. Midguts of whiteflies exposed to TYLCV-infected tomato plants for a 3 d AAP were dissected and prepared for immunofluorescence. Blue signal indicates the cell nucleus. Green signal indicates TYLCV. Red signal indicates early endosomes (Rab5), late endosomes (Rab7) or recycling endosomes (Rab11). Scale bar 5 μm. (TIF) [file ppat.1006866.s009.tif]

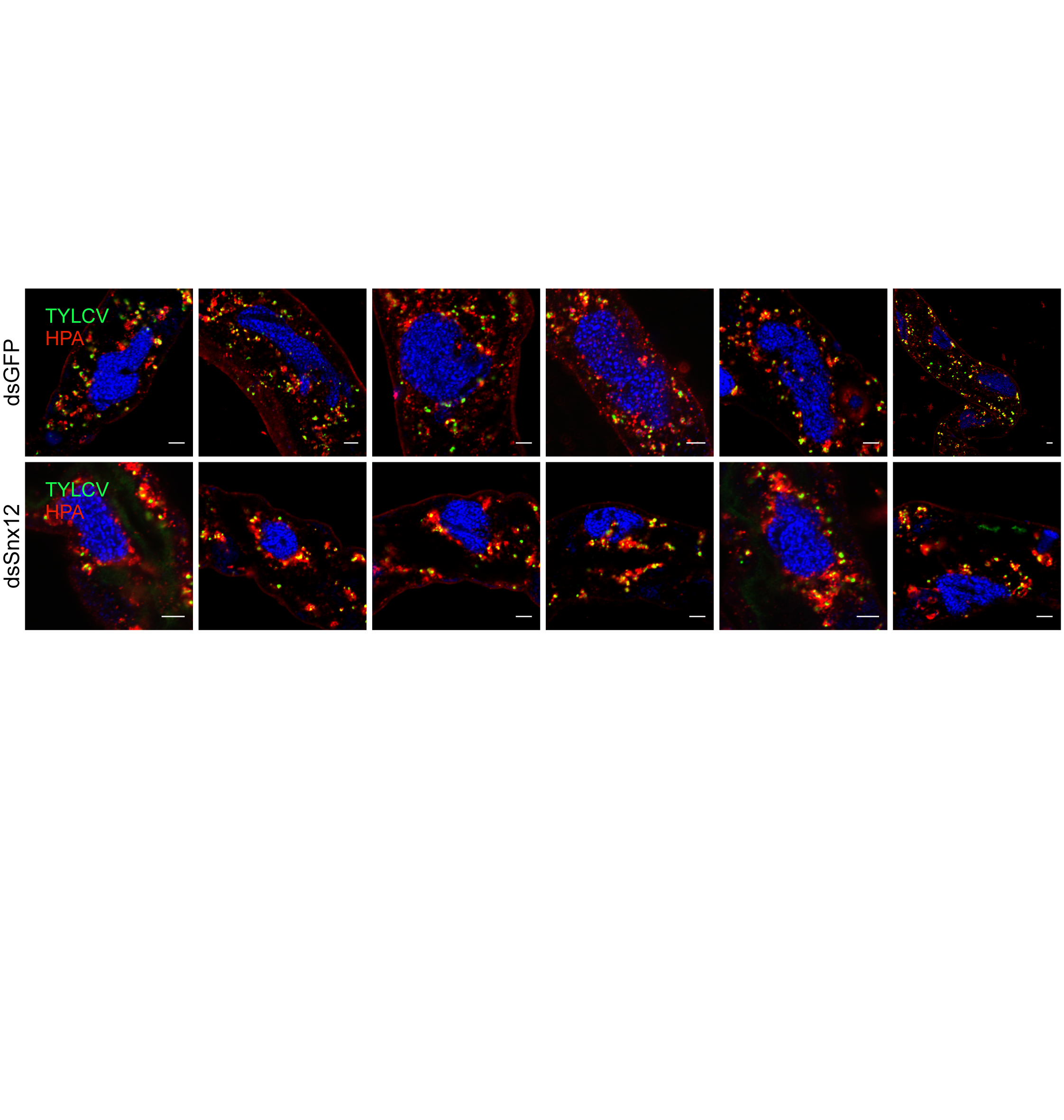

Supplement: S10 Fig — Representative images that were used to generate Pearson’s coefficient in Fig 10. Three days after dsRNA injection, whiteflies were allowed to feed on TYLCV-infected plant for three days and prepared for immunostaining. Blue signal indicates the cell nucleus. Green signal indicates TYLCV. Red signal indicates labelling of HPA lectin. Scale bar 5 μm. (TIF) [file ppat.1006866.s010.tif]

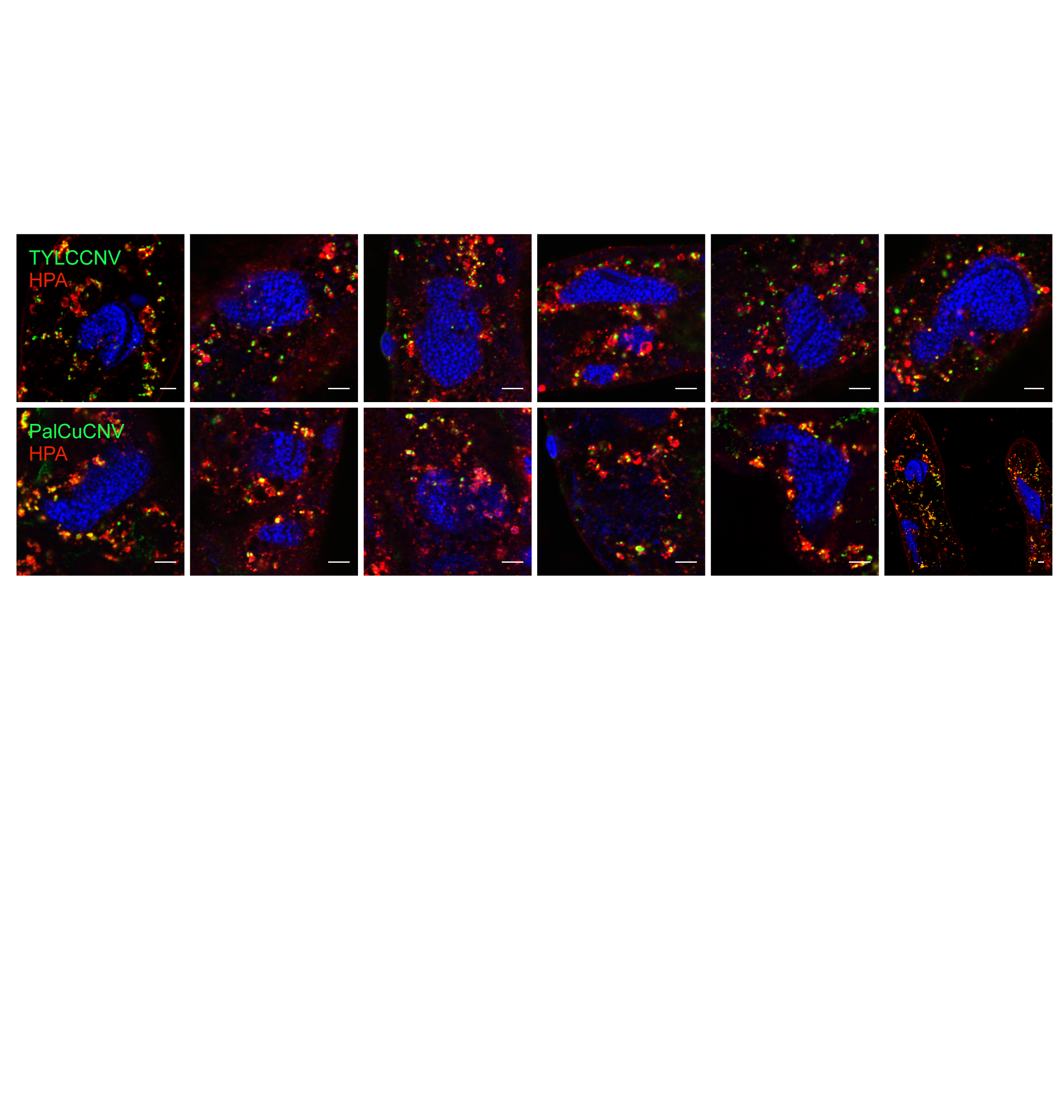

Supplement: S11 Fig — Representative images that were used to generate Pearson’s coefficient in Fig 11. Midguts of whiteflies exposed to PalCuCNV- or TYLCCNV-infected tomato plants for a 3 d AAP were dissected and prepared for immunofluorescence. Blue signal indicates the cell nucleus. Green signal indicates PalCuCNV or TYLCCN. Red signal indicates labelling of HPA lectin. Scale bar 5 μm. (TIF) [file ppat.1006866.s011.tif]
